# Molecular Mechanism of EGFR-TKI Resistance in *EGFR*-Mutated Non-Small Cell Lung Cancer: Application to Biological Diagnostic and Monitoring

**DOI:** 10.3390/cancers13194926

**Published:** 2021-09-30

**Authors:** Damien Reita, Lucile Pabst, Erwan Pencreach, Eric Guérin, Laurent Dano, Valérie Rimelen, Anne-Claire Voegeli, Laurent Vallat, Céline Mascaux, Michèle Beau-Faller

**Affiliations:** 1 Department of Biochemistry and Molecular Biology, Strasbourg University Hospital, CEDEX, 67098 Strasbourg, France; Damien.REITA@chru-strasbourg.fr (D.R.); erwan.PENCREACH@chru-strasbourg.fr (E.P.); eric.GUERIN@chru-strasbourg.fr (E.G.); laurent.dano@chru-strasbourg.fr (L.D.); valerie.rimelen@chru-strasbourg.fr (V.R.); anne-claire.VOEGELI@chru-strasbourg.fr (A.-C.V.); laurent.VALLAT@chru-strasbourg.fr (L.V.); 2 Bio-imagery and Pathology (LBP), UMR CNRS 7021, Strasbourg University, 67400 Illkirch-Graffenstaden, France; 3 Department of Pneumology, Strasbourg University Hospital, CEDEX, 67091 Strasbourg, France; Lucile.PABST@chru-strasbourg.fr (L.P.); celine.MASCAUX@chru-strasbourg.fr (C.M.); 4 INSERM U1113, IRFAC, Strasbourg University, 67000 Strasbourg, France

**Keywords:** *EGFR* mutations, non-small cell lung cancer, tyrosine kinase inhibitors, resistance mechanisms, molecular analysis, next-generation sequencing, cell-free DNA

## Abstract

**Simple Summary:**

Non-small cell lung cancer (NSCLC) is the most common cancer in the world. For common *EGFR* mutations, treatment is based on different inhibitors. Despite the excellent disease control with inhibitors, acquired resistance inevitably occurs and remains a biological challenge. This leads to the discovery of novel biomarkers and some possible drug targets. Resistance mechanisms could be involved as gene mutations, amplifications or fusions, which could be detected by different molecular techniques on different types of biological samples. Histological transformation is another mechanism of resistance with some biological predictive factors that needs tumor biopsy. The place of liquid biopsy also depends on the generation/line of inhibitors and could be a good candidate for molecular monitoring. This article is based on the literature and proposes actual and future directions in clinical and translational research.

**Abstract:**

Non-small cell lung cancer (NSCLC) is the most common cancer in the world. Activating epidermal growth factor receptor (*EGFR*) gene mutations are a positive predictive factor for EGFR tyrosine kinase inhibitors (TKIs). For common *EGFR* mutations (Del19, L858R), the standard first-line treatment is actually third-generation TKI, osimertinib. In the case of first-line treatment by first (erlotinib, gefitinib)- or second-generation (afatinib) TKIs, osimertinib is approved in second-line treatment for patients with T790M *EGFR* mutation. Despite the excellent disease control results with EGFR TKIs, acquired resistance inevitably occurs and remains a biological challenge. This leads to the discovery of novel biomarkers and possible drug targets, which vary among the generation/line of EGFR TKIs. Besides *EGFR* second/third mutations, alternative mechanisms could be involved, such as gene amplification or gene fusion, which could be detected by different molecular techniques on different types of biological samples. Histological transformation is another mechanism of resistance with some biological predictive factors that needs tumor biopsy. The place of liquid biopsy also depends on the generation/line of EGFR TKIs and should be a good candidate for molecular monitoring. This article is based on the literature and proposes actual and future directions in clinical and translational research.

## 1. Introduction

Lung cancer remains the most common cause of cancer deaths worldwide. The molecular classification of non-small cell lung cancers (NSCLCs) leads to the discovery of oncogenic drivers, which could be targetable. Among them, the most frequent is represented by activating epidermal growth factor receptor (*EGFR*) gene mutations, and several generations of EGFR tyrosine kinase inhibitors (EGFR-TKIs) are now available. Sensitivity to EGFR-TKIs depends on the type of *EGFR* mutations, and the development of resistance to such TKI inevitably occurs. The identification of these resistances remains challenging. Here, we propose an extensive review of the literature about molecular mechanisms of EGFR-TKI resistance in EGFR-mutated NSCLC, with a focus on biological diagnostic and monitoring, including the place of new molecular technologies and liquid biopsies.

**Figure 1:**
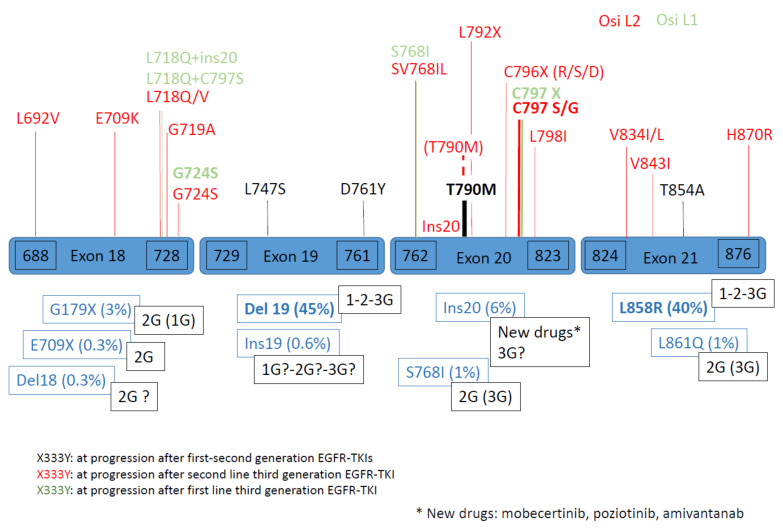
*EGFR* mutations.

EGFR (Figure 1) is one of the ERBb family of receptor tyrosine kinases with four members: EGFR (ERBb1/HER1), ERBb2/HER2/NEU, ERBb3/HER3 and ERBb4/HER4. Specific ligands bind to the extra-cellular domain of EGFR, which leads to the formation of homodimers and heterodimers. Dimerization stimulates intrinsic tyrosine kinase activity of the receptors and triggers the auto-phosphorylation of specific tyrosine kinase residues. Signal transducers initiate multiple downstream pathways, such as MAPK, PI3K-AKT and STAT 3 and 5, which regulate cell proliferation and apoptosis.

### 1.1. Molecular Epidemiology of EGFR Mutations

*EGFR* exons 18 to 24 encode the tyrosine kinase domain of EGFR, and *EGFR* activating mutations are located in four exons (18, 19, 20, 21) (Figure 1). These mutations are responsible for the constitutive activation of EGFR and consequently, the activation of several pathways, which lead to cell proliferation, among others. Their frequency is around 10 to 15% in Caucasian non-small cell lung cancers (NSCLC) and 30 to 50% in Asian NSCLC [1,2]. The *EGFR* mutation frequency could vary among populations, from less than 10% to more 50%, and is classically more important in adenocarcinoma, nonsmokers, women or Asian NSCLC patients [1].

The first-line treatment of choice for patients with *EGFR* activating mutations NSCLC is based on EGFR tyrosine kinase inhibitors (EGFR-TKIs), such as gefitinib, erlotinib, afatinib, dacomitinib and osimertinib. Most of the clinical trials with EGFR-TKIs concern only patients with common *EGFR* mutations (exon 19 deletion, exon 21 L858R point mutation) which account for 90% of mutations [3,4,5,6,7]. Besides common *EGFR* mutations, uncommon *EGFR* mutations account for 10% of mutations. They are represented by rare *EGFR* mutations and complex *EGFR* mutations. Complex *EGFR* mutations are multiple *EGFR* mutations in a same sample, identified in one or several of four exons, 18 to 21. If common *EGFR* mutations are sensitive to EGFR-TKIs, uncommon *EGFR* mutations present variable predictive values, due to the different effects on the tertiary structure of EGFR proteins. Co-mutations are defined by *EGFR* mutation(s) combined with at least one mutation of another gene (oncogene or tumor suppressor gene), driver or not.

Classification of *EGFR* mutations depends on their type as substitutions (change of one or more nucleotides or punctual mutation), deletion (del) of nucleotides, insertions (ins) of nucleotides, or insertions combined with deletion (indel). Substitutions could also be combined with other type of mutations. The localization of amino acids is also useful to classify *EGFR* mutations, from 688 to 728 for exon 18, 729 to 761 for exon 19, 762 to 823 for exon 20 and over 824 for exon 21 (Figure 1). The international classification is based on nucleotides and/or amino acids. Nevertheless, three factors appear to complicate the estimation of the true frequencies of each *EGFR* mutation in clinics: molecular methods, the presence of complex mutations, and publications’ biases.

#### 1.1.1. Common EGFR Mutations: Deletion in Exon 19 (Del19) and Exon 21 Point Mutation, L858R

Structural studies have shown that Del19 and L858R mutations destabilize the inactive conformation of EGFR receptor, leading to increased receptor dimerization and activity [4]. L858R lies within the helical turn of the activation loop and forms crucial hydrophobic interactions with residues in the N-lobe. The L858R substitution locks the kinase domain in a constitutively active conformation. Del19 shorts the β3-αC loop, which prevents the outward rotation of the αC-helix [4].

Large data are available concerning common *EGFR* mutations in metastatic NSCLC. In such cases, Del19 (44–51%) appears more frequently than L858R (38–40%) in Asian as well as in Caucasian populations. Some clinical characteristics could be different, as the proportion of current/former smokers is significantly lower in Del19 and L858R, compared to other *EGFR* mutations (*p* = 0.02) [3]. *EGFR* mutations are prognostic with better progression-free survival (PFS) and overall survival (OS) under first–second-generation EGFR-TKI or third-generation EGFR TKI for Del19, compared to L858R mutation [3,8,9,10].

With new indications of EGFR-TKIs in an adjuvant setting (ADAURA study) [11], it is interesting to analyze *EGFR* mutation patterns in stages I–III NSCLC patients. In such cases, *EGFR* mutations are more frequent in stage I (20 to 50%), compared to stages II or III (5 to 20%) [12,13]. The mutation of L858R (48.5%) was more frequent than del 19 (40.8%) in one recent Asian retrospective study [14]. Recurrence free-survival (RFS) and OS are better in cases of *EGFR* mutation positive cases [14]. The clinical impact of *EGFR* mutation subtypes in early-stage NSCLC are described, with EGFR Del19, compared to L858R, being associated with younger onset, larger consolidation size, higher frequency of pure-solid tumors, significant correlation with higher pathological stage and poorer PFS [14].

If L858R mutation corresponds to one type of substitution (c.2573T>G, p.L858R), del19 covers more than 30 variants. The different Del19 variants start at position 746 with a lot of variants that delete LRE amino acids; the most frequent deletion is that of delE746_A750 (73%), with a deletion of 9 to 24 nucleotides. Some other Del19 variants begin at position E747 (25%). Other Del19 variants are described as entitled non LRE (2%). Some of these deletions are not detected by molecular kits and could be associated with few insertions; delins are rarer (>1%).

Besides common *EGFR* mutations, uncommon *EGFR* mutations are described, of which molecular diagnosis is facilitated by new molecular techniques, such as next-generation sequencing (NGS) or multiplex polymerase chain reaction (PCR), in FFPE tumor samples as well as in liquid biopsy, which is easy to practice and able to detect *EGFR* mutations in plasma.

#### 1.1.2. Rare EGFR Mutations

Rare *EGFR* mutations are represented by *EGFR* mutations other than common *EGFR* mutations (exon 19 deletion, exon 21 L858R point mutation) (Figure 1). More than 600 *EGFR* different mutations are actually described in the Catalogue Of Somatic Mutation in Cancer (COSMIC) database with a variable or unknown biological impact and/or EGFR-TKI sensitivity [4,8,15]. Rare *EGFR* mutations could be classified as “rare” mutation, such as some *EGFR* exon point mutations of 10% (exon 18, G719X; exon 20, S768I; exon 21, L861Q) and “rarer” *EGFR* mutations for the others [3,16]. Their clinical presentation is not different, compared to common *EGFR* mutations, except for CNS metastases, which appear more frequently in uncommon mutations (54%) [17,18,19,20,21,22,23]. Mechanisms of resistance to EGFR-TKIs are described most of the time for common EGFR mutations.

### 1.2. Secondary and Tertiary EGFR Mutations

Secondary and tertiary *EGFR* mutations are defined as mutations that are highlighted after treatment by EGFR-TKIs. The most classical secondary *EGFR* mutation after first/second-generation EGFR-TKIs is T790M mutation (50%). After third-generation EGFR TKI osimertinib used in second-line treatment for T790M mutation, tertiary *EGFR* mutations, such as L718Q (exon 18), C797X, L792X, C796X, L798I, ins20 (exon 20), for example, can be detected. After third-generation EGFR TKI osimertinib is used in first-line treatment, secondary complex *EGFR* mutations, such as L718Q + L797S (exon 18 + 20), L718Q + ins20 (exon 18 + 20), Del19 + G724S (exon18), C797X or S768I (exon 20), can be detected [4,15,16].

### 1.3. Complex EGFR Mutations

Complex *EGFR* mutations represent a heterogenous group of mutations with a prevalence estimated to be between 5 and 15% of all EGFR mutations. They are composed by combined common and uncommon EGFR mutations, uncommon and uncommon *EGFR* mutations or common and common *EGFR* mutations. G179X is involved in more than 90% of complex mutations, G709X in more than 75%, and S768I in more than 50%. Pre-clinical data show variable sensitivity to EGFR TKIs, depending on the different combinations, with resistant complex mutations (T790M + L858R), variable sensitivity (E709A + G719C, Q797R + L858R, H870R + L858R, E884K + L858R) and sensitive mutations (E709A + G719C, G787R + L858R, H870R + L858R, E884K + L858R). The sensitivity depends on the associated mutation and is better with Del19 or L858R (best with Del19 + L858R), and lower with sensitivity mutation combined with resistance mutation. The sensitivity of complex mutation including exon20ins is variable; double ins20 could be sensitive to EGFR-TKIs.

### 1.4. Co-Mutations

Many EGFR-mutant tumors harbor one or more co-mutations depending on the used next-generation sequencing (NGS) panels (size and coverage) for molecular analysis [24]. Combined recurrent mutations are found in *TP53*, *CTNNB1 (β-catenin)*, *RB1* or *PI3KCA* as well as some gene amplifications [25]. The spectrum and prevalence of co-mutations are similar across the three most common EGFR mutations (Del19, L858R, and ins20). Prior treatment appears to be associated with an increased number of such co-alterations. Mutations in *PIK3CA* or *CTNNB1* are more frequent in advanced-stage tumors, whereas mutations in *TP53*, *Rb1* and *NKX2-1* appear to occur in early-stage and advanced-stage tumors. *TP53* mutations are a negative prognostic marker in EGFR-mutations NSCLC and a predictor of worse clinical outcomes under EGFR-TKI therapy [25,26]. *TP53* and *Rb1* co-mutations could transform to small-cell carcinoma following exposure to EGFR-TKIs although loss of RB1 is insufficient to directly induce neuroendocrine differentiation.

*PTEN*-inactivating mutations, *ATM* alterations, and *IDH1* mutations are predictors of short PFS in patients receiving first-generation EGFR-TKI; *PTEN*, *ATM*, *IDH1* and *KRAS* mutations as well as alterations in the MAPK pathway are related to short OS in the French Biomarker Study [27].

### 1.5. Sub-Clonal Mutations

Some tumors present “subclonal” mutations with a low variant allele (VAF) frequency, suggesting that the mutation appears only in a small proportion of tumor cells [28]. These sub-clonal mutations could be detected for all types of *EGFR* mutations, and particularly in complex *EGFR* mutations and for resistant *EGFR* mutations, such as T790M mutation. The T790M clonality level may influence the response to third-generation EGFR-TKI. Retrospective analysis of the AURA study led to the characterisation of patients who were T790M-positive only in plasma, suggesting that the mutation is only present in a fraction of tumor cells in these patients [29]. This subset of patients showed the shortest PFS with a lower objective response rate. Patients who had lost the T790M mutation at progression had significantly shorter PFS and tended to have a smaller fraction of T790M related to the activating *EGFR* mutations in their tumor at baseline [30]. In the AURA3 clinical study, subclonal T790M (VAF < median as 30%) analyzed by NGS was associated with shorter PFS in patients treated by osimertinib [31]. These samples were enriched for *PI3KCA* mutations, which reduces sensitivity to osimertinib in vitro [31]. Another study demonstrated the presence of T790M mutation analyzed by ddPCR in 8% of pre-treatment EGFR-mutated NSCLC samples, which has an independent prognosis value, which depends on VAF [32].

The tertiary structure of EGFR protein, which is affected by different mutations, combined with different generation EGFR-TKIs (structural, biochemical), could explain the different sensitivities to EGFR-TKIs of different *EGFR* mutations. For uncommon mutations, pre-clinical results on NSCLC cells are available, using different molecular technology as an exogenous expression of rare *EGFR* mutants in different model cell lines [4,15]. Structural and preclinical data were used to predict the efficacy of different EGFR-TKIs for specific, rare *EGFR* mutations. There are very few clinical trials that systematically and robustly evaluate the efficacy of EGFR-TKIs in NSCLC patients that harbor rare *EGFR* mutations. Due to the paucity of clinical data, the field is largely reliant on pooled post hoc analysis of clinical trials and case series to evaluate the response of EGFR-TKIs in this heterogeneous group of patients.

## 2. Mechanisms of Resistance to EGFR-TKIs

### 2.1. Clinical Trials Results

EGFR TKIs were tested in order to become the new treatment standard for advanced EGFR mutation–positive NSCLC patients. EGFR-TKIs alone were tested through different clinical trials (Table 1). New studies are now also available of EGFR-TKIs combined with other usual NSCLC treatments.

#### 2.1.1. First Line Therapy of NSCLC with EGFR Mutation

##### First-Generation EGFR TKI

First-generation EGFR-TKIs (erlotinib, gefinitib, and icotinib) are reversible TKIs, which reversibly bind to the ATP-binding pocket of EGFR. Not very efficient in unselected advanced NSCLC, first-generation EGFR TKIs were found to have a high efficacy for patients with advanced NSCLC tumors harboring *EGFR* mutations. Since the first-line treatment for those patients was initially platinum-based combination chemotherapy, EGFR TKIs were compared to chemotherapy agents in second- then in first-line treatments (Table 1) [33,34,35,36,37,38,39]. When progression-free survival (PFS) was increased in all groups of patients treated by EGFR-TKIs, there was no statistical difference in OS.

##### Second-Generation EGFR-TKIs

Second-generation EGFR-TKIs (afatinib and dacomitinib) are irreversible pan-HER (EGFR, HER2 and HER4) and were developed to overcome *EGFR* T790M mutation. Despite the promising pre-clinical data, the concentration of the drug did not reach the treatment range for T790M because of relatively severe adverse events, compared to first-generation EGFR-TKIs, due to the inhibition of wild-type EGFR. However, afatinib and dacomitinib are approved as first-line treatments for patients with *EGF*R mutations [40,41,44,45,46]. When PFS was increased in all groups of patients treated by EGFR-TKIs, there was no statistical difference in OS. The post hoc analysis of LUX-Lung 2, 3 and 6 indicated that afatinib was especially active in some uncommon *EGFR* mutations, such as G719X, S768I and L861Q, while it was less effective for patients with de novo T790M mutations and exon 20 insertion mutations (Yang JC, July 2015) [45].

##### Third-Generation EGFR-TKIs

The pyrimidine-based third-generation TKI was developed, targeting T790M mutation as well as common EGFR mutations but without inhibiting wild-type EGFR. Osimertinib in first-line treatment for patients with *EGF*R common mutations, compared to standard EGFR-TKIs (gefitinib or erlotinib), demonstrated longer PFS as well as overall survival (OS) than those who received a comparative EGFR-TKI in the FLAURA study [9,10]. Other third-generation EGFR-TKIs (rociletinib, olmutinib, and nazartinib) still need more clinical evaluations.

##### EGFR Ins20 Specific Inhibitors

Specific inhibitors of the EGFR ins20 (poziotinib and mobocertinib) were developed, as these mutations are not sufficiently sensitive to first–second–third-generation EGFR-TKIs. Poziotinib showed clinical activity in patients with EGFR/HER2-exon-20-ins-mutation but with high rates of adverse events leading to treatment discontinuation or posology reductions that underline the necessity of further trials [47]. Mobocertinib showed a more favorable toxicity profile in preclinical studies and phase I/II trials [42]. A bispecific monoclonal antibody, amivantanab, targeting EGFR and MET, inducing immune-directed antitumor activity, showed promising results [43].

##### EGFR-TKI Treatments Combinations

*EGFR-TKI Combined with Targeted Therapy*

Double inhibition of EGFR using EGFR TKI plus EGFR monoclonal antibody (cetuximab) did not show any improvement in terms of PFS for afatinib + cetuximab versus afatinib alone [48].

*EGFR-TKI Combined with Antiangiogenic Molecules*

Bevacizumab is a vascular endothelial growth factor (VEGF) monoclonal antibody that inhibits angiogenesis, which plays a crucial role in tumor proliferation and metastasis. Some phase II studies compared the efficacy of the association erlotinib–bevacizumab to erlotinib alone and concluded the longer median PFS with the combination treatment [49,50]. Ramucirumab, a human IgG1 VEGFR2 antagonist, in association with erlotinib, did not seem to prevent the emergence of the T790M mutation, but may delay its appearance [51].

*EGFR-TKI Combined with Chemotherapy*

Platinum-based chemotherapy combined with erlotinib showed prolonged PFS and OS with improved ORR, compared to chemotherapy (Wu Q) [52]. Pemetrexed combined with gefitinib, and compared with gefitinib alone, showed better PFS and overall response rate (ORR) without benefit to OS [53]. Preclinical results with third-generation TKI osimertinib combined with pemetrexed or cisplatin also showed delay in acquired resistance and long-lasting effects, even after treatment discontinuation, but osimertinib administered before chemotherapy was less effective [54,55]. A question that arises is about the antagonist effect of EGFR-TKIs when administered before chemotherapy by a TKI-induced G1-phase blockade, which then protects cells from chemotherapy toxicity. Phase III of the FLAURA2 study is currently ongoing and is testing the association of chemotherapy to osimertinib.

*EGFR-TKI Combined with Radiotherapy*

Both treatments separately induce an accumulation of tumor cells in the G(1) and G(2)-M phases and a decrease in cells in the S-phase; the association enhances the reduction in cells in the S-phase by radiosensitization, inducing cell cycle arrest and apoptosis. The association of thoracic radiotherapy and EGFR-TKI for metastatic EGFR-mutant NSCLC showed a long-term control of the primary tumor with better PFS than with EGFR-TKI alone [56]. The continuation of EGFR-TKI after local progression with concurrent radiotherapy also demonstrated better ORR and local tumor control rate [57], warranting further studies. Some contradictory results suggest that this combination should be used with caution, even in cases with brain metastasis.

#### 2.1.2. Second-Line with Third-Generation EGFR-TKIs

After confirming the benefit in ORR with osimertinib in the AURA1 trial [58], the phase I/II AURA2 trial [48,59] demonstrated higher ORR and median PFS among patients with T790M secondary mutation combined with common *EGFR* mutation. The AURA3 study finally concluded with the superiority of osimertinib over platinum-pemetrexed chemotherapy for patients previously treated with EGFR-TKI and harboring a T790M mutation [60]. Osimertinib is nowadays a standard-care treatment for patients with *EGFR* T790M mutation.

### 2.2. Mechanisms of Resistance

Despite the clinical activity of first–second–third generation of EGFR-TKIs, 5–25% of NSCLC patients with a tumor harboring EGFR-activating mutations do not respond to these targeted therapies. In such situations, primary resistance occurs. After an initial response (complete response or partial response) or stable disease, patients inevitably develop secondary resistance, which poses a significant challenge to detect the mechanism of resistance for the treatment of such resistance. The preexistence of resistant clones and the development of adaptative resistance define innate/primary and acquired/secondary resistance.

Another situation corresponds to the development of resistance in tumor cells subpopulations within a generally sensitive tumor. In fact, complete tumor response is rare and incomplete tumor response (identified by conventional radiographic imaging or occult disease) is followed by therapy-resistant tumor progression, illuminating the problem of residual disease. The biological mechanisms underlying the occurrence of residual disease in patients at the time of maximal initial-therapy response remain poorly understood, mostly owing to the lack of direct analysis of samples from patients with residual disease and the lack of cancer models that faithfully recapitulate human tumor responses [61].

EGFR-TKIs treatment can fail because of drug resistance, a lack of drug target or inadequate drug exposure. Mechanisms of resistance depend on drug efficacy on the target, drug efficacy on the adaptative mechanisms, and drug-induced mutability. The identification of resistance mechanisms is essential, but is currently based on clinical cases or small series of clinical assays with no modeling.

The molecular heterogeneity of NSCLC tumors could influence the possible mechanisms of resistance to EGFR-TKIs, contributing to the wide spectrum of resistance aberrations. Multiple co-existing molecular alterations were observed in a considerable percentage of patients, for whom osimertinib was administrated as a second-line or front-line therapy as well as in the cases of the failure of previous EGFR-TKIs.

**Figure 2:**
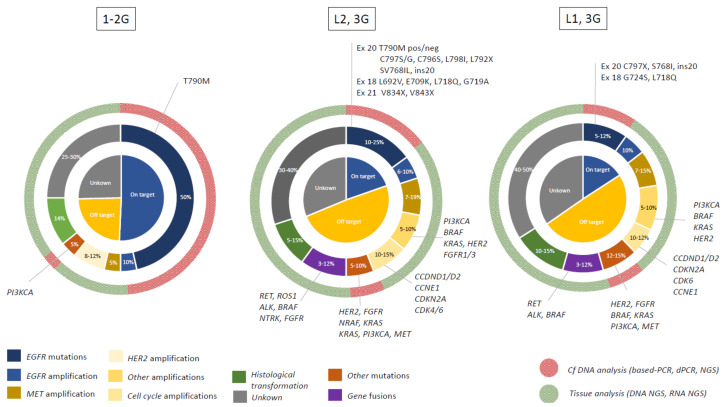
Mechanisms of resistance to EGFR-TKIs, depending on the generation of TKIs and on the line of therapy; 1–2G :first–second-generation EGFR-TKI; 3G:third-generation EGFR-TKI; L1:first line of treatment; L2:second line of treatment.

**Figure 3:**
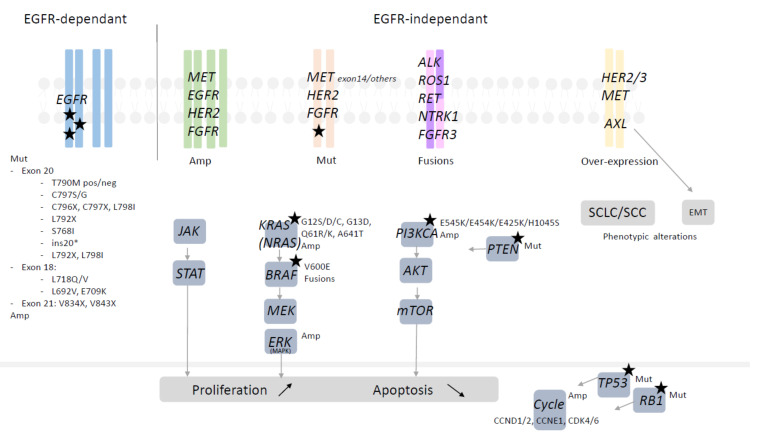
EGFR signaling pathway and EGFR-TKIs’ acquired resistance mechanism.

As third-generation EGFR-TKI osimertinib is currently used in first-line treatment for common EGFR-mutated NSCLC patients, we focus on the mechanism of acquired resistance after such treatments [10]. Nevertheless, acquired resistance to first/second-generation EGFR-TKIs created the area of understanding the molecular and histological mechanisms of resistance, with T790M *EGFR* mutation as the dominant mechanism of resistance [62,63]. Resistance to third-generation EGFR-TKI osimertinib in second-line therapy allowed comprehension of such situations [64,65] (Figure 2 and Figure 3). The characterization of resistance enables the development of subsequent therapies [64,65,66,67,68].

The main first studies of osimertinib resistance are based on ctDNA analysis, for AURA3 in second-line treatment as well as for FLAURA in first-line treatment [58,69]. Other cases of acquired resistance mechanisms are often based on little series or numerous case reports. Furthermore, some published studies have lacked paired tumor samples, pre- and post-osimertinib, which makes determination of the acquired alterations and putative resistance mechanism challenging. One recent study was based on paired tumor tissues to detect all molecular as well as histologic mechanisms of resistance to osimertinib and identify potential associations with clinical outcomes in small groups of patients (after second-line osimertinib, *n* = 35; after first-line osimertinib, *n* = 27) [70].

Mechanisms of acquired resistance seem recently to be more common than the selection of preexisting drug-resistant sub-clones [71].

Finding predictive biomarkers of resistance has important implications for NSCLC care. Initial *EGFR* mutation (common *EGFR* mutations Del19 or L858R versus exon 20), the generation of EGFR-TKIs and the line of therapy all greatly influence the resistance spectra identified.

#### 2.2.1. Primary Resistance

Primary resistance corresponds to a rare situation of intrinsic or innate resistance before any EGFR-TKIs administration, with early tumor progression without prior tumor response. The response rate and disease control rate are effectively not at 100% but are around 75–80% for EGFR-TKIs, regardless of the generation of drugs; some patients respond for a very short duration (< 3 months) [10,72,73]. A particular situation of primary resistance is pharmacokinetic therapy failures that result in incomplete drug impact [61]. This situation is observed in cases of drug competition or with some sanctuary localization, such as the brain, for first/second-generation EGFR-TKIs [62].

Primary resistance is mostly related to the lack of a target dependency (i.e., *EGFR* exon 20 mutations) or the molecular alterations of genes from other pathways (downstream or parallel pathways).

##### Primary Resistance to First–Second-Generation EGFR-TKIs

The presence of *EGFR* T790M mutation at diagnosis is a rare event that suggests, in some cases, germinal *EGFR* mutation [74]. In such cases, first- or second-generation EGFR-TKIs are not efficient [75]. The presence of pre-treatment T790M mutation is reported widely, with highly variable incidence rates (< 1–65%) depending on the sensitivity of the molecular tests and related to the worst outcomes [76,77].

Finally, *MET* amplification in naïve EGFR-TKIs is a rare event with incidence in less than 5% of patients (Turke). Co-mutations of the TP53 gene detected in plasma within two months of EGFR-TKI treatments is a negative prognostic factor for PFS and OS [78]. The under-expression of BIM or NF1 is a bad prognosis factor for PFS [79,80], as is the overexpression of RhoB [81].

Some alterations can coexist with *EGFR* mutation at baseline and are associated with bad response to first-generation EGFR-TKIs, such as *AXL* and *CDCP1* RNA overexpression [82]. AXL is a receptor-kinase and was previously implicated in epithelial–mesenchymal transition (EMT) (Figure 2). Co-alterations in other genes of the MAPK, PI3K and Wnt/β-catenin pathways or cell cycle genes were associated with poor response to EGFR-TKIs [24,73].

Contrary to the mutual exclusivity of the majority of oncogenic driver mutations, the co-occurrence of PI3KCA mutations with some other oncogenic driver mutations is well described in NSCLC [83]. The impact of concurrent *PI3KCA* mutations with *EGFR* mutations is not evident in clinical outcomes of patients treated by EGFR-TKIs [84]. The co-occurrence of *EGFR* Del19 with non-disruptive *TP53* exon 8 mutations is associated with primary resistance to first-generation EGFR-TKIs [85].

##### Primary Resistance to Third-Generation EGFR-TKIs

Except for EGFR T790M mutation, primary resistance mechanisms to third-generation EGFR-TKIs are generally comparable to those for first–second-generation EGFR-TKIs. Nevertheless, some particularities are noted. Osimertinib showed excellent central nervous system (CNS) penetration in preclinical studies as well as in clinical trials, both as first-line and second-line treatments in patients with EGFR-mutant NSCLC [58,69] with no CNS sanctuary.

Data are mostly based on primary resistance to second-line osimertinib [66]. The identification of primary resistance in second-line treatment could depend on the area of biological testing realized at the time of resistance after first–second-generation EGFR-TKIs. For example, SCLC transformation was described as a putative mechanism of primary resistance to osimertinib in five cases, only tested in cfDNA by ddPCR for T790M analysis [86]. In such cases, a low ratio (lower than 0.03) between T790M and EGFR-activating mutation in the blood was detected before osimertinib treatment. For three patients, EGFR mutational analysis was T790M-negative when re-assessed by using a less sensitive method (Therascreen) on the same liquid biopsy sample analyzed by ddPCR before osimertinib therapy.

*MET* amplification could represent a potential mechanism of intrinsic resistance to osimertinib [87,88]. *HER2* amplification was reported, as HER2 overexpression decreased sensitivity to osimertinib and rolecitinib in vitro [66,87,88].

*KRAS* G12D mutations combined with PTEN loss were also detected in patients with primary resistance to second-line osimertinib [73]. Some alterations can coexist with EGFR mutation at baseline and are associated with bad response to third-generation EGFR-TKIs, such as AXL and CDCP1 RNA overexpression [82].

#### 2.2.2. Secondary Resistance

All the patients develop secondary or acquired resistance with progression after an initial response or stable disease to first/second-generation EGFR-TKIs. The clinical criteria of acquired resistance are well defined [87,88]. The biological mechanisms of secondary resistance are challenging, depending on the tumor biology (*EGFR* mutation, co-mutations, intrinsic mutability, microenvironment and histological transformation), drug (pharmacology, inhibition of adaptative mechanisms, mutability), generation of EGFR TKIs and the line of EGFR-TKI treatment.

Acquired resistance mechanisms to EGFR TKIs can be classified into EGFR-dependent mechanisms and/or EGFR-independent mechanisms. Some of the mechanisms are overlapping depending on the generation of EGFR-TKIs and/or line therapy, whereas others were identified only in one of these settings (Figure 2 and Figure 3).

Acquired resistance to first/second generation EGFR-TKIs usually appears after 9–12 months of therapy. Acquired resistance to third-generation EGFR-TKIs used in second-line treatment still occurs after about 10 months [60]. Acquired resistance to third-generation EGFR-TKIs in first-line treatment occurs after about 19 months [10].

While numerous studies of resistance at progression under first–second-generation or second-line third-generation EGFR-TKIs after developing T790M mutation are available, studies of resistance to first-line osimertinib treatment are less frequent. As more and more patients with *EGFR*-mutated NSCLC tumors will be treated with this new paradigm, it is important to progress our understanding of resistance in this context. In a study with paired tissues, concurrent genomic alterations were identified in 71% (25/35) cases after second-line osimertinib treatment and in 41% (11/27) of cases after first-line osimertinib treatment [70].

##### EGFR-Dependent Mechanisms of Resistance

The relative incidence of *EGFR*-dependent or on-target–dependent mechanisms of resistance differ, according to the generation of TKI used and the line of therapeutics. Patients receiving first–second-generation EGFR-TKIs predominantly (50%) develop on-target resistance, compared to 20% for third-generation TKI as second-line treatment and 10–15% for first-line treatment [89].

*EGFR* mutations/amplification arise quickly and are located in critical amino acid residues. They induce conformational changes of the kinase (gate-keeper mutations), cause direct steric hindrance by limiting drug accessibility to the kinase ATP-binding pocket (solvent-front mutations) or by increasing the ATP affinity of the mutant *EGFR*.

*To First–Second-Generation of EGFR-TKIs*

Half (49–63%) of the patients develop EGFR T790M mutation (exon 20) at the time of progression under first–second-generation EGFR-TKIs, with a subset of these patients also developing *EGFR* amplification, with the T790M allele being specifically amplified [63]. This secondary T790M point mutation is an exon 20 substitution resulting in steric hindering to the binding of first–second-generation EGFR-TKIs and increasing receptor affinity for ATP binding, with a consequent drastic reduction in drug activity without affecting drug affinity itself. Other rarer acquired EGFR mutations are described as D761Y and L747S (exon 19) or T854A (exon 21) [74,90,91].

Other molecular alterations could also be detected in association with *EGFR* T790M mutation, such as *β-catenin* mutation [63].

*EGFR* amplification is detected in 8–10% of patients, all of which have T790M mutation [63]. EGFR amplification could also be detected with L858R pre-treatment samples, with MET amplification and loss of EGFR amplification at the time of resistance [63].

There appear to be few differences in the acquired resistance biological mechanisms between first- and second-generation EGFR-TKIs [91]. For example, EGFR C797S mutations and low frequency of T790M mutations are described in cell-free DNA (cfDNA).

*To Third Generation of EGFR-TKIs*

Analysis of the literature suggests that second-line and first-line osimertinib treatment present different resistance spectra to *EGFR* mutations.

A. In second line

Second-line osimertinib treatment is used in tumors harboring *EGFR* T790M mutation, suggesting that tumors have continued dependance on EGFR signaling and may be predisposed to acquiring tertiary *EGFR* mutations. The first results of the mechanisms of resistance at progression under second-line osimertinib therapy come from cell-free DNA (cfDNA) genomic profiles from NSCLC of the AURA3 trial [69,92]. Half of the patients retained the T790M mutation, comprising the totality of patients with tertiary EGFR mutation. Acquired third EGFR mutation was reported in 21% of cases, with *EGFR* exon 20 C797S mutation in the majority of cases (15%). C797S accounted for 10–26% of other cases of resistance to second-line EGFR-TKI [60,93]. C797S occurred in EGFR exon 20 as T790M mutation, with a cysteine of the ATP-binding site substituted by a serine in position 797. This modification resulted in the loss of the covalent bond between osimertinib and the mutant *EGFR* by binding interference [94]. In a tumor study, EGFR C797S frequency was higher for 29% of cases and was preferentially coupled with EGFR exon 19 deletion compared to L858R (24% versus 11%), and was only seen in tumors that retained T790M, suggesting continued EGFR dependance in these tumors [70]. C797S mutation confers cross-resistance to other irreversible third-generation EGFR-TKIs (rociletinib, olmutinib and narzatinib) by preventing their binding to the EGFR active site [95,96]. The rare cases (less than 30%) in which C797S is located in *trans* (on different alleles) with the T790M mutation, cells could be targeted with both first–second- and third-generation EGFR-TKIs [97,98]. On the contrary, when the mutation C797S is in *cis* with the T790 mutation (on the same allele), the cells were found to be resistant to all EGFR-TKIs, alone or combined [96,98].

In one study with 24% of C797S third *EGFR* mutation, co-existing C797G mutation was anecdotally detected by NGS in cfDNA samples in two cases [98]. C797G mutation was also detected by NGS in one case with a MYC and EGFR concomitant amplification pleural sample [99]. In a tissue study, C797 mutations were a common occurrence in 29% (10/35) of cases, with C797S (*n* = 9) or C797G (*n* = 1), being always associated with retained T790M mutation [70].

Besides C797X mutations, other rare point EGFR mutations were identified as solvent front mutations in the C796 residue (G796R, G796S, G796D), adjacent to C797 in exon 20, with the potential to sterically interfere with the osimertinib EGFR interaction. G796R had a major impact, compared to C796S on osimertinib-EGFR binding [95,100,101]. Another G796D mutation was detected [102].

L792 residue (L792H) is located in the “hinge” region of the kinase, and can sterically interfere with a methoxy group of osimertinib by disrupting its binding to the kinase domain [91,101]. L792H mutation can occur in *cis* with T790M, but also in *trans* with other EGFR mutations, such as C796/C797X. These L792 mutations remain sensitive to gefitinib in vitro [98].

Another rare tertiary EGFR mutation with binding interference L798I was also been described [91].

Substitution of 718 residue located in the ATP-binding site of the EGFR kinase domain can cause spatial restriction for binding osimertinib [98]. They are represented by L718Q. Another mutation L718V was reported in a clinical case with loss of T790M [103]. Patients with L718 mutations generally do not have co-existing C797 mutations, suggesting that these mutations could lead independently to osimertinib resistance. The G719A mutation is close to the L718 residue and also causes osimertinib resistance [98]. The L718Q mutation might still be sensitive to first–second-generation EGFR-TKIs, especially in cases with loss of T790M [65,104].

G724S mutation (exon 20), located in exon 20 in the P-loop of the EGFR kinase domain, was also identified [105,106]. It impairs the binding of osimertinib and preferentially occurs with EGFR exon 19 deletion, but not L858R, in an allele-specific manner. In the absence of T790M mutation, second-generation TKIs retain kinase affinity with successful in vitro activity [107].

Other mutations within EGFR exon 20 rarely occur. SV768IL (S768I + V769L) rare mutation (3%) was detected in second-line treatment with osimertinib [70]. Exon 20 insertion was reported in one patient (1%) [69]. It was also detected at baseline with varied sensitivity to EGFR TKIs.

More often, third *EGFR* mutations are combined with maintained T790M, in position *cis*, conferring resistance to all first/second/third-generation EGFR-TKIs [96]. All patients with third *EGFR* mutations retained the T790M mutation in the AURA3 study. In the tissue study, the presence of the *EGFR* T790M mutation was enriched in the samples with an acquired third *EGFR* mutation (*p* = 0.04), but *EGFR* SV768IL was described in this last study with loss of T790M [70].

The amplification of *EGFR* in addition to exon 19 deletion is the mechanism of resistance described in cfDNA [108]. Increased EGFR mRNA expression was also described [109]. In the cfDNA analysis of the AURA3 study, *EGFR* amplification was not described. *EGFR* amplification (6%) was described in the tissue study with loss of T790M mutation [70] and was found to correspond to wild-type *EGFR* amplification.

However, near half (43%) of the patients lost T790M mutation at progression [30,65,69,103,109]. Loss of T790M suggests that EGFR T790M mutation exists as a subclone [89]. In such cases, exon 19 deletion (83%) was preferentially present, compared with L858R (14%) mutation [69]. Loss of T790M mutation at the time of progression is usually associated with early resistance to osimertinib and a shorter time to treatment discontinuation (60 versus 15.2 months) [30]. Further studies confirmed the negative impact of T790M loss on PFS and OS [69,109,110]. Loss of T790M is usually associated with loss of EGFR dependence and dependance of non-EGFR mechanisms [69]. In rare cases, the emergence of a third EGFR mutation could occur in cases of loss of T790M mutation, with, for example, L718Q, G724S, V834L mutations [65] or L718V [103]. These last cases appear particularly interesting, as acquisition of a third EGFR mutation alone without T790M mutation might be overcome by quinazoline-based first/second-generation EGFR-TKIs [65]. Plasma levels of T790M mutation and activating EGFR mutations could predict the type of acquired resistance mechanism [30].

B. In first line

In the FLAURA trial in which NSCLC patients received osimertinib in first-line treatment, NGS of cfDNA samples did not show any emergence of T790M mutation, as expected by the pharmacodynamics activity of osimertinib, selective for both EGFR-sensitizing and T790M mutations [58]. In this trial, the on-target mechanism with *EGFR* mutation/amplification was rare (9%). C797S mutation frequency was 7%, lower than in the second-line setting but making it, nevertheless, the most frequent mechanism, behind *MET* amplification. Other secondary *EGFR* mutations are very rare (1–2%), such as S768I (1%) or combined *EGFR* mutation, such as L718Q + C797S (1%) or L718Q + EGFR ex20ins [58]. *EGFR* amplification is not included in this cfDNA study. In keeping with these data, on-target resistance was rare in the tissue study, with 8% of cases having EGFR mutation/amplification. Only one acquired G724S (4%), with no C797S among 27 patients and only one (4%) *EGFR* amplification [70]. Notably, the paucity of the on-target resistance mechanism after first-line osimertinib treatment in this tissue study may be a function of the short follow-up (median time on osimertinib of 13.6 months in this study and 18.3 months in FLAURA), suggesting a possible bias toward early progressors [111]. Thus, the overall spectrum of resistance may change with a longer follow-up.

Alternatively, these findings my reflect a difference in biology between first- and later-osimertinib use. Patients who receive osimertinib as a second-line treatment have already developed *EGFR* T790M mutation, demonstrating a predilection for on-target resistance mechanisms, and could, therefore, be more able to develop other EGFR alterations at progression upon second-line osimertinib treatment.

As osimertinib is swiftly moved to first-line treatment, the incidence of T790M mutation as a resistance mechanism will become less frequent, despite it remaining one of the on-target resistant mechanisms.

##### EGFR-Independent Mechanisms of Resistance

MET receptor tyrosine kinase signaling is the most frequently altered pathway involved in EGFR resistance following EGFR-TKI, irrespective of the EGFR-TKI generation or line of treatment. The occurrence of *MET* mutations or increased ligand HGF (hepatocyte growth factor) is rare, and the MET-mediated resistance mechanism occurs via *MET* amplification. The result is bypass EGFR signaling via MAPK, PI3K or STAT pathways (Figure 3).

*To First–Second-Generation of EGFR-TKIs*

*MET* amplification is reported in 5–22% of cases, preferentially with EGFR exon 19 deletion [63,112]. There is a lack of consensus on the definition of MET amplification; different techniques can be used with discordant findings. The most widely adopted definition for *MET* amplification is the presence of the *MET* gene with a copy number of ≥5 or a MET/CEP7 ratio of ≥2 [113].

The *HER2* gene encodes the ErbB2 receptor tyrosine kinase. It mediates EGFR TKI resistance through alternative activation via MAPK or PI3K pathways. *HER2* amplification was detected in 12% of tumor samples at progression after first-generation TKI, with no co-existing T790M mutations [114]. As for *MET* amplification, variable criteria have been developed for *HER2* amplification with different NGS analysis, in tissue or plasma.

Other rare gene mutations, such as *BRAF, KRAS, β-catenin* (< 1%) are described. Bypass activation of the PI3K pathway can occur via both mutation/amplification of *PI3KCA* and *PTEN* deletion. *PI3KCA* mutations and *PTEN* loss are responsible for increased PI3K signaling [91]. *PI3KCA* mutations are known to co-occur at baseline with some other driver mutations in NSCLC. Concurrent *PI3KCA* mutation with *EGFR* mutation do not impact the clinical outcome of patients treated by EGFR-TKIs [84] or are related to a shorter PFS in such patients [63]. *PI3KCA* (3–5%) mutations or amplification are observed at progression after first-generation EGFR-TKI, but these mutations may also have been combined already at diagnosis with *EGFR* mutations, demanding its role in acquired resistance. Insulin-like growth factor 1 receptor (IGF1R) was shown to bypass gefinitib blockage of EGFR signaling via activation of the PI3KCA pathway [91].

Upregulation of the AXL gene with overexpression of the protein was also reported in acquired resistance to EGFR-TKIs [91,115].

Gene fusions are not impossible but very rare; gene fusions are described at progression after first–second-generation TKIs. For example, two cases with *BRAF* fusions at progression after erlotinib were recently described [116]. This low frequency could be due to modification of treatments with third-generation TKIs administrated at first-line treatment in *EGFR*-mutated patients, and to the low detection of gene fusion at progression after first-second generation TKIs.

Histologic transformation from NSCLC to small cell lung cancer (SCLC) is a known mechanism of resistance to first- and second-generation EGFR-TKIs (14%) and was first described in 2011 [63]. The original EGFR mutation was maintained. No case with squamous cell transformation was described. The underlying mechanism of such a transformation is still missing. Mutations of *RB1* and *TP53* genes could be potential predisposing factors, with inactivation of these tumor suppressor genes found in the initial NSCLC as well as in SCLC at progression, as was found in de novo SCLC [117,118]. Co-mutation of *EGFR*, *TP53* and *RB1* is a key risk factor for eventual transformation, with a 43-fold increased risk of transformation. Nevertheless, the biologic mechanisms that drive such histological transformation remain poorly understood. EMT could also be described (8%) with changes in vimentin and E-cadherin expression [63]. SCLC and EMT represent less-common mechanisms of acquired resistance.

*To Third Generation of EGFR-TKIs*

The activation of alternative pathways and/or histologic transformation are other mechanisms of resistance. They can co-occur in the same tumor and co-exist with *EGFR* tertiary mutation EGFR-independent (off-target) mechanisms, including histologic transformation, which emerges earlier, resulting in a less durable response to osimertinib. On the other hand, EGFR-dependent mechanisms (on-target mechanisms), i.e., T790M, could be associated with more indolent disease after a longer time on EGFR-TKIs with better post-progression survival [78].

A. In second line

Besides data based on 73 matched pre/post-osimertinib cfDNA analysis in the AURA3 trial, one recent study presented a series of 35 matched pre-post treatment biopsies obtained from patients with EGFR-mutant NSCLC treated with osimertinib in second-line therapy [69,70].

*MET* amplification constitutes the most frequent cause of bypass pathway activation. *MET* amplification can occur with or without loss of T790M mutation. In the AURA3 trial, *MET* amplification was observed in nearly (14/73) 19% of the cfDNA samples at progression. *MET* amplification co-occurred with C797S EGFR mutation in 7% of cases, as well as *CDK6* or *BRAF* amplification [65,69]. A single genomic event of chromosome 7 was hypothesized, as *MET*, *CDK6* and *BRAF* are located in 7q31, 7q21 and 7q34, respectively [65]. *MET* amplification was reported at a lower rate (6%) in tumor tissues [70]. It seems difficult to provide a conclusion on *MET* amplification real frequency because there is no consensus on the definition of *MET* amplification by NGS analysis using liquid biopsy. On one hand, *MET* amplification could be overestimated when lacking pre-treatment tumor analysis, and on the other hand, cfDNA platforms typically have lower sensitivity to assess copy number changes [65,69,119,120,121,122,123,124,125]. *MET* amplification was seen concurrently with *EGFR* mutation prior to treatment [65,120].

Rare *MET* mutations (P97K/Q, I865F) were identified in the cfDNA of patients with second-line osimertinib treatment [95]. MET exon 14 mutations were also detected in tissue analysis in 3% of patients in such a situation [70].

*HER2* amplification was identified in 5% of patients after second-line osimertinib treatment, mutually exclusive with T790M mutation (as was found after first-generation EGFR-TKIs) [114,126]. *HER2* amplification was described in cfDNA in (4/73) 5% of cases, co-existing with T790M mutation, *PI3KCA* amplification or gene cycle amplification (1%) [69]. In a tissue study, *HER2* mutation (Y772_A775dup) (3%) was described in a case of retained T790M mutation, but no HER amplification [70].

*NRAS* mutations (and novel E63K) were described in vitro together with a gain of a copy of wild-type *NRAS* or wild-type *KRAS* in NSCLC EGFR–mutated cell lines resistant to gefitinib, afatinib or osimertinib [127]. The in vitro and in vivo combination of osimertinib with selumintinib, a MEK inhibitor, were able to prevent EGFR-TKI resistance. *KRAS* mutations were described but are very rare events. Mutation *KRAS* G12S was described in the case of resistance after second-line osimertinib treatment [87]. Other *KRAS* mutations, such as G12D, G13D, Q61R and Q61K, were also identified at progression after osimertinib in second-line treatment in less than 1% of cases [30,62,65,69]. *KRAS* mutation (G12D) (3%) was described in a tissue study in cells lacking T790M [70]. No targetable *KRAS* G12C mutation was described at progression under second-line osimertinib. *BRAF* V600E mutations were identified as a resistance mechanism at progression after second-line treatment in 3% (3/73) of cases in cfDNA [69] in association, or not, with T790M mutation [109,128]. In vitro *BRAF* V600E NSCLC cell lines as a resistance mechanism to osimertinib showed sensitivity to an association with osimertinib and a BRAF inhibitor (encorafenib) [128]. *MAPK1* mRNA overexpression was described in one patient at progression after osimertinib second-line treatment [109].

The role of *PI3KCA* E545K mutation in osimertinib resistance was confirmed in vitro [65]. At progression after osimertinib second-line treatment, *PI3KCA* mutations (E454K, E452K, R88Q, N345K, E418K) were described in 4–11% of patients [30,65,69,129]. Amplification of *PI3KCA* was described by NGS analysis of cfDNA in AURA3 study with co-existing *HER2* amplification in two out of three cases [69]. The frequency of PI3KCA mutation reached 17% in a tissue study [130]. The loss of PTEN was also described as an acquired mechanism [130].

Several studies have reported the amplification of other genes (*cyclin D1, cyclin D2, cyclin E1, cyclin-dependent kinase N2A, CDK4/6*) or frameshift deletion of *CDK inhibitors*. Alterations of gene regulating cell cycle were described in 12% of cases after second-line osimertinib treatment in cfDNA [69].

Other rare EGFR-independent events can occur with alterations in the *FGFR* family (amplifications) and with alterations in signaling pathways, such as the *Src* family kinases or *AXL* receptor tyrosine kinase receptor [89] (Figure 3).

Gene fusions. They are oncogenic drivers with potential targeted treatment and are described in 3–10% of cases after second-line osimertinib treatment and can co-occur with *EGFR* C797S mutation, *BRAF* mutation or *MET* amplification [69]. The most frequently reported are *RET* fusion (46%), followed by *ALK* (26%), *NTRK1* (16%) and *FGFR3* (11%) [131]. They are represented by *RET* fusions (*RET-ERC1, RET-CCDC6, RET-NCOA4*), *ROS1* fusions (*ROS1-GOPC*), *BRAF* fusions (*BRAF-AGK, BRAF-ESYT2, BRAF-PCBP2, BRAF-BAIAP2L1, BRAF-PJA2*) [30,64,65,116]. Another gene fusion with *ALK* (*ALK-EML4*) was described after second-line osimertinib treatment [132]. In a tissue study, *ALK* fusions (6%) were described with retained T790M and *BRAF* fusions (3%) with loss of T790M [70]. *NTRK1* fusion (*NTRK1-TPM3*) was described in combination with Del19 and T790M *EGFR* mutations in cfDNA analysis. *FGFR3* fusions were also described (*FGFR3-TACC3*) combined with *EGFR* Del19, T790M and C797X mutations [69]. More recently, a *MET* fusion (*MET-SPECC1L*) was described in cfDNA [133]. As opposed to the classic oncogenic drivers in naïve NSCLC tumors, they frequently harbor uncommon 5’ partners, such as *NCOA4* for *RET* fusion or *CCDC6* for *ALK* fusions, with unusual breakpoints.

Histologic and phenotypic transformation.Histologic and phenotypic transformation could be expected only with tissue analysis and was not described in the AURA3 study with cfDNA analysis [69]. As described for first-second generation EGFR-TKIs and with the same frequency (14%), histologic transformation from NSCLC to SCLC is a known mechanism of resistance to third-generation EGFR-TKIs in second-line osimertinib treatment. The original EGFR mutation was maintained. Histologic transformation from NSCLC to small cell lung cancer (SCLC) is a known mechanism of resistance to third-generation EGFR-TKIs in second-line osimertinib treatment [70,97,134]. Histological transformation occurred in 5 of 35 (14%) patients, with 3 (8%) to SCLC. This high frequency could have implications for future research as well as for clinical practice. A serial whole-genome sequencing of SCLC-transformed tumors demonstrated that the clonal origins of SCLC-transformed cells were distinct from T790M-resistant cells that also emerged in the same patients [118]. Histologic transformation toward three squamous cell carcinoma (9%) was also observed at progression after second-line treatment [70]. Similarly, as for SCLC transformation, *EGFR* mutation was maintained.

B. In first line

In addition to the data based on 61 matched pre/post-osimertinib cfDNA in the FLAURA trial, one recent study presented a series of 27 matched pre-post treatment biopsies obtained from patients with EGFR-mutant NSCLC treated with osimertinib in first-line therapy [58,70]. The FLAURA series showed the on-target resistance mechanism to be rare after first-line osimertinib, but was notably limited by its inability to assess for some other non-target resistance mechanisms, such as histologic or phenotypic transformation, and by the low sensitivity of NGS analysis on cfDNA for the detection of amplification. Schoenfeld studies were based on tissue analysis but were limited to a smaller number of patients as well as to early progressors. This last study identified acquired resistance mechanisms in 41% (*n* = 11/27) of cases.

When osimertinib was given in first-line treatment, *MET* amplification was the most common resistance mechanism (15%) in cfDNA analyzed by NGS [58]. This percentage could be higher in tumor tissues, due to the underestimation of amplification by NGS in ctDNA. Nevertheless, *MET* amplification was related in only 7% of cases in tissue study [70]. Several pre-clinical studies demonstrated that the concomitant use of MET inhibitors (crizotinib) with osimertinib could overcome such resistance [65,87,135]. *MET* mutation (MET H1094Y) (3%) was recently described in a patient after failure of first-line osimertinib [70]. No *MET* mutation was found in the FLAURA biological cfDNA analysis [58].

After osimertinib first-line treatment, *HER2* amplification was detected in 2% of cases analyzed in cfDNA, coexisting or not with other EGFR-dependent or -independent resistance mechanisms [58]. *HER2* amplification was not described in a tissue study [70].

*KRAS* mutations, such as G12D/C or A146T (3%), were described in FLAURA [58]. KRAS G12A mutation was also described in one case (4%) in a tissue study (Schoenfeld). *BRA*F V600E mutations (3%) were identified as a resistance mechanism at progression after first-line osimertinib [58]. No *BRAF* mutation was described in a tissue study (Schoenfeld). Two cases of *BRAF* V600E mutation combined with MET amplification were described after first-line osimertinib [58,86].

*PI3KCA* mutations (E453K, E545K and H1047S) were described at progression under first-line oismertinib in six cases (7%), with E545K being the most represented (4%) [58]. No *PI3KCA* mutation was described in the tissue study [66].

Alterations of the gene regulating cell cycle are described in 10% after first-line treatment [58] with amplification of *CCND1/D2* (3%), *CCNE1* (2%), and *CDK4/6* (5%) genes and are associated with poor outcomes.

Gene Fusions. Just one fusion involving *ALK* (*ALK-SPTBN1*) was described in one patient (1%) in cfDNA after first-line osimertinib [58]. Another study identified another *ALK* fusion (*ALK-PLEKHA7*) following osimertinib treatment [136]. In a tissue study, no *ALK* fusion was described, but others targeted such fusions as *BRAF* (*BRAF-TRIM24*) or *RET* (*RET-RUFY2*) in 4% of cases [70].

Histologic and Phenotypic Transformation. Histologic and phenotypic transformation could be expected only with tissue analysis and was not described in the FLAURA study [58]. Histologic transformation from NSCLC to small cell lung cancer (SCLC) is a known mechanism of resistance to third-generation EGFR-TKIs, as well as to first-line osimertinib treatment [70]. Histological transformation occurred in 9 of 27 (15%) patients, with 3 to SCLC. This high frequency could have implications for future research as well as for clinical practice. Histologic transformation toward squamous cell carcinoma was also observed at progression after first-line osimertinib with 5 cases among 9 patients with histological transformation [70]. The biological type of squamous cell transformation is even less characterized, compared to SCLC transformation. Despite broad genomic analysis, there were no common molecular features among the 5 patients with squamous cell carcinoma, and there were no putative biomarkers that could be used to identify patients at highest risk of such transformation. It is common for NSCLC to have a mixed histology with adenocarcinoma and squamous cell carcinoma. Squamous transformation could just reflect a shift in the predominant histological type rather than a true lineage shift. Patients with squamous cell carcinoma had short post-progression survival.

Phenotypic transformation as epithelial-to-mesenchymal transformation (EMT) was reported in NSCLC tumors of patients with acquired resistance to osimertinib, with a decrease in epithelial proteins, such as E-cadherin, an increase in mesenchymal proteins, such as vimentin (Weng CH, Oncogene 2019) and in osimertinib-resistant cells, an upregulation of EMT transcription factors, such as the zinc finger Zeb1 [137] or TWIST-1 [138].

#### 2.2.3. Particular Points

##### Comparison Second/First Line Osimertinib

The first-line frequency of C797S was lower, compared with second-line treatment, suggesting that first-line and second-line osimertinib may have different resistance spectra. EGFR C797S, the most common EGFR mutation after second-line osimertinib treatment, was not identified in first-line treatment in the tumor study [70]. In this tissue study, the proportion of off-target and unknown resistance emerged earlier and were higher in the first-line than in the second-line setting (*p* = 0.01) [70].

##### Other Rare Mechanisms

The amplification of *FGFR1* and overexpression of *FGF2* at the mRNA level were reported at progression under second-line osimertinib treatment in one patient, suggesting an autocrine loop-mediated mechanism [109]. One case of *FGFR3/FGF19* amplification was also described at progression under second-line osimertinib [65].

##### Variability of Resistance Mechanism Depends on Type of EGFR Mutation

Recent data suggest that the initial sensitizing *EGFR* mutations may create bias in the resistance mechanisms that emerge. In a tissue study, post-osimertinib treatment *EGFR* C797S mutation was more frequently seen with *EGFR* Del19 than L858R mutation (24% versus 11%, respectively), and *CDKN2A/B* deletion and *TERT* amplifications were more commonly seen with L858R mutations, compared to *EGFR* Del19 (*p* = 0.02 and *p* = 0.03, respectively) [70]. In this study, *EGFR* G724S was only identified with *EGFR* Del19 at resistance after first-line osimertinib. Structural and in vitro models supported *EGFR* G724S as conferring resistance only when concurrent with an *EGFR* Del19 [107]. Other gene amplifications, gene fusions and non-*EGFR* mutations seem comparable between common *EGFR* mutations [69,70] but they are represented by few cases to validate such a sub-group analysis. In a tissue study, histological transformation seemed more frequent with *EGFR* Del19 (17%) rather than with L858R (5%, only squamous), but not significantly [70]. No robust data are available about resistance mechanism with rare uncommon *EGFR* mutation, particularly after osimertinib treatment, or with complex *EGFR* mutations, because they are not in the field of third-generation EGFR-TKIs treatment and are also represented by a large number of little sub-groups of *EGFR* mutations [139]. On another hand, no robust representative data are available concerning resistance mechanisms depending on the presence of co-mutations at baseline.

##### Resistance Treatments

It is not clear whether rare *EGFR* mutations, such as S768I, which are described de novo and have mixed response to first/second EGFR TKIs, will also respond to earlier generation TKIs when acquired as on-target resistance to osimertinib. By contrast, acquired *MET* mutation H1094Y can be overcome in vitro by combined inhibition of MET (crizotinib) and EGFR (osimertinib) [70]. Tumors harboring gene fusions previously described in naïve NSCLC tumors, even concerning unusual partners and breakpoints, seem to respond or control disease under treatments targeting the gene, such as for *ALK, RET* fusions, usually with maintaining EGFR-TKI [132]. The situation appears different in cases with *BRAF* fusions in which BRAF inhibitors were not effective and could require combined MEK and EGFR inhibition [116].

Histological transformation, which cannot be detected by plasma analysis, is a frequent event. Rates of transformation and other off-target resistance mechanisms may be higher with osimertinib, compared with earlier generation EGFR-TKIs, due to better on-target inhibition. Contrary to SCLC, squamous cell transformation does not have an overarching signature, and requires further gene expression analysis as well as a non-genomic process, such as transcription factor network or epigenetic analysis.

*EGFR* T790M mutation is the most common resistance mechanism to first- and second-generation EGFR-TKIs, but resistance mutations in genes, such as *MET*, *PI3KCA*, and *NF1,* are also often found; these can be present in distinct cell populations [24]. Genes in the PI3K pathway (*PI3KCA)* or cell cycle pathways (*CCNE1*), are the most frequently altered TKI-resistance genes in the T790M subclonal tumors. One study recently showed that *PI3KCA* mutation (H1047R) emerges in a small fraction of cells and provides a growth advantage under osimertinib treatment in vitro, independently of T790M mutation [31]. Such sub-clonal levels of PI3KCA mutations, which could be acquired during previous lines of therapies with first–second-generation TKI, might have important therapeutic implications.

##### Gene Silencing

EGFR-TKIs must be ineffective in the case of gene silencing, when *EGFR* mutations lack protein expression [140].

##### Other Situations of Resistance

A recent study related mechanisms of resistance after association of savolitinib and osimertinib in cases with amplification of MET at resistance under osimertinib. Whole exome analysis revealed MET-dependent mechanisms of resistance, such as acquired *MET* mutation (D1246H, Y1230C in kinase domains of MET) and *MET* copy number gain. As for MET-independent mechanisms, the development of *HER2* mutation and amplification, mutations in *KDM5C*, *ARAF*, *NFATC2* and copy number gains in genes involved in cell-cycle machinery (*CCNE1*, *CCND1*, *CDK6*) [89].

There is no resistance mechanism described under new EGFR therapeutics, such as mobocertinib or amivantanab.

Evidence supporting different resistant mechanisms according to the treatment line suggests the potential variability of such mechanisms in adjuvant situations [11].

#### 2.2.4. Perspectives

Comprehensive genetic profiling of the tumor tissue at disease progression leads to identifying resistance mechanisms in order to select the most appropriate therapeutic combination approach. In fact, one of the main objectives to characterize the specific resistance mechanism is to detect targetable molecular alterations, such as mutations or gene fusions for biomarker-driver therapies. Nevertheless, different phase II trials combining osimertinib with specific TKIs, according to the identified pattern of resistance in preclinical models, are ongoing [89]. Furthermore, the biomarkers’ results depend on the type of sample or type of molecular methods used for testing. The results are actually focused on the resistance mechanism in tumors harboring common EGFR mutations, such as Del19 and L858R, and no consistent resistance mechanisms are available for uncommon or complex *EGFR* mutations.

Combinations of EGFR TKIs with other treatments are currently under investigation to delay resistance by targeting subclones, which could emerge under selective pressure. Next-generation TKIs with more potent and irreversible binding properties, avoiding the ATP-competitive TKI binding to mutated domains, delay the occurrence of resistance. Fourth generation of EGFR-TKI (overcoming C797S and T790M mutations) have demonstrated in vitro and in vivo activity, alone or in combination with osimertinib.

**Figure 4:**
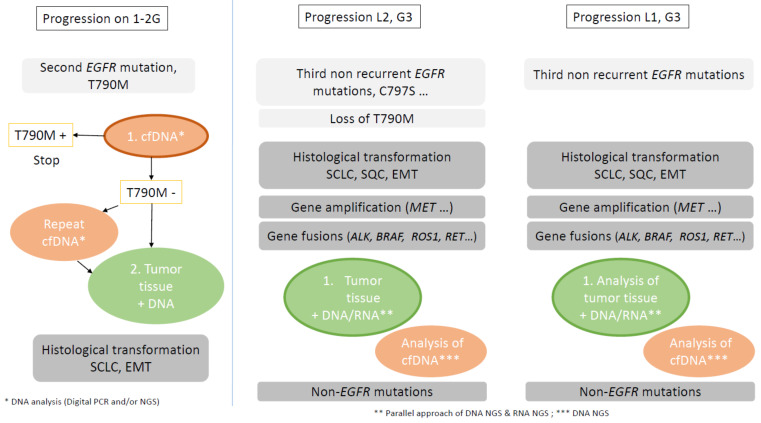
Strategy for molecular analysis at progression under EGFR-TKIs.

Understanding the dynamics of the different alterations associated with EGFR resistance and the interplay with the different lines of therapy will help to guide clinical decisions. The presence of *TP53* mutation or co-occurrence with *RB1* mutations and the lack of plasma clearance of *EGFR* mutations are mechanisms that could help to identify patients with no durable response under EGFR TKIs. The place of cfDNA monitoring remains to be assessed. cfDNA analysis has the potential to detect the mutation of time and/or spatial heterogeneity, but has an inability to detect easier gene fusions or amplifications and is unable to detect histological transformation (Figure 4).

## 3. Technical Aspects of Molecular Testing

As described above, the resistance mechanisms underlying EGFR-TKI resistance are multiple; molecular alterations include single nucleotide variations, small duplication/insertions or deletions (indels), gene copy number variations and gene fusions in numerous genes. The identification of these various but also rare molecular alterations at resistance requires an enlarged molecular testing in clinical routine.

For genomic clinical laboratories, the sequential analysis of multiple genes/molecular alterations is expensive, time consuming and requires large amounts of tumor tissue and nucleic acids. In this context, multiplex techniques, allowing the analysis of multiple alterations in samples from several patients at the same time, are taking an increasingly important place in analysis strategies [51,141] (Table 2).

### 3.1. Nucleic Acids DNA, RNA and TNA

DNA- and RNA-sequencing remain the current reference methods for the detection of all types of mutations. It can be used to detect gene amplifications based on the observed sequencing depth data, but it is recommended to confirm these observations by another validated method, such as fluorescence in situ hybridization (FISH) or digital PCR. Indeed, classical molecular sequencing techniques are often not sensitive enough to detect such alterations, in cases of low levels of amplification, low content of tumor cells or when using cell-free DNA (cfDNA).

Immunohistochemistry (IHC) can be used as a screening method to detect expressed fusion genes. However, this method relies only on qualitative scoring and the availability of high-quality antibodies. FISH has long been the gold standard for detecting chromosomal rearrangements, but it lacks scalability for high throughput multi-target testing. Neither IHC nor FISH provide information on the exact fusion partner breakpoint [142]. Detection of gene fusions should be analyzed preferentially by RNA sequencing–based techniques. RNA-based approaches have the dual advantage of allowing the analysis of only transcriptionally expressed fusion genes and determining the exact breakpoint and fusion partner. DNA sequencing–based techniques are hampered by the presence of intronic sequences, which are sometimes very long and repetitive, decreasing the performance of gene fusion detection and increasing dramatically the cost of sequencing [143,144].

In addition, RNA gene panels, besides gene fusions detection, allow a simultaneous analysis of expression levels of genes with a clinical value [145]. Nevertheless, even though RNA sequencing–based targeted approaches are more accurate than DNA panels for tumor tissue analysis, they can be limited by RNA quality and quantity [146]. Moreover, the extraction of RNA from formalin-fixed, paraffin-embedded (FFPE) tissue samples is tricky, and the failure rate of RNA sequencing is about 10 to 20% depending on the method used [147].

Biopsy material is precious, and it is often difficult to obtain large amount of material or separate tissue sections. Besides the use of distinct extraction kits for DNA and RNA, commercial kits are now available to isolate both DNA and RNA (TNA, total nucleic acid) from the same starting material, sparing precious specimens [148,149]. In addition to preserving material, the great advantage of TNA is to use, in the same assay, RNA for gene fusion and oncogenic isoforms detection and DNA for single nucleotide variants, indels and copy number variations detection [145,146,150].

Cell-free DNA (cfDNA) can be an alternative to tumor biopsy [151]. However, because cfDNA is highly fragmented and its concentration can be extremely low against a high background of normal circulating DNA, screening for clinically relevant mutations can be challenging. The major drawback of cfDNA analysis is the very high rate of false negative results, which is why, in the case of a negative result, it is often recommended to continue the analysis by performing a tissue biopsy, which may identify a molecular alteration despite a negative result of the cfDNA [29,152]. Furthermore, cfDNA is more performant in detecting mutations, compared to other molecular alterations, such as copy number variation (CNV) or gene fusions.

### 3.2. Molecular Analysis

If historical molecular analysis of tumor samples is sequential, using multiple Sanger sequencing and/or other PCR-based molecular techniques, two global multiplex strategies have emerged to analyze tumor genomic material at progression [153]: comprehensive and high-throughput methods based on next generation sequencing (NGS), or targeted non-NGS methods based on the characterization of only well-known and predefined alterations. NGS analysis appears to be the best molecular technique for detecting the various alterations described at progression. Furthermore, the choice of molecular technique may be different from a clinical perspective, compared to a research analysis (Table 2).

#### 3.2.1. Next-Generation Sequencing (NGS)

Whole genome sequencing (WGS) or whole exome sequencing (WES) are extensive analyses of the genome with identification of all known or unknown mutations. These technologies require a large amount of biological material and are not compatible with FFPE samples. Their mean coverage appears low with decreasing sensitivity of the techniques. Whole transcriptome sequencing (WTS), or RNAseq, allows extensive analysis of known and unknown expressed gene fusions. These RNA techniques are more sensitive than DNA analysis, but the quality and quantity of RNA from clinical samples may be inadequate. In fact, in clinical practice, neither WGS nor WTS are routinely used since these methods are expensive, time consuming and require a complicated data analysis workflow [154]. The clinical utility of these extended tests has to be evaluated, particularly for resistance situations [155].

As a more focused sequencing approach, targeted NGS panels are the most used in the clinic, with specific commercial panels developed by pathology, until larger “pan-cancer” panels targeting a large number of alterations or custom design panels become available. With this approach, only genomic regions of interest are sequenced, simplifying downstream bioinformatics analysis, reducing costs, and offering the ability to obtain greater depth of coverage, improving confidence in the base, calling for variant analysis and detection threshold [156,157].

For DNA-targeted NGS sequencing, the choice of method depends on the advantages of each method and includes a variety of parameters, such as cost, amount of sample input required, sensitivity, and specificity. Many commercial kits are available as well as targeted house panels. Although RNA-targeted NGS sequencing can also be based on hybrid-capture or amplicon-based methods, most studies used the latter ones for transcript fusion detection [158]. Numerous commercial kits are available, targeting from ten (FusionPlex kit Archer^®^, Oncomine Focus Thermo Fischer) to one hundred genes (TruSight RNA fusion panel Illumina, Targeted RNAscan custom Qiagen), with amplicon-based approaches or anchored PCR. The amplicon-based approach does not represent the best technique in the case of progression under EGFR-TKI, due to the large number of partners and possible break points. By contrast, anchored PCR allows the detection of all possible fusion variants of a gene included in the panel by using specific primers binding to common adapters that are covalently linked to the unknown sequences and is highly sensitive [159,160]. The failure rate of RNA analysis from FFPE sample for gene fusions detection was from 10 to 20% in a recent study, mainly because the quality of RNA was too poor in some samples [161].

The analysis of cfDNA can also be done by targeted NGS [162]. Mutations, CNV and gene fusions detection, covering the full spectrum of molecular mechanisms of EGFR-TKI resistance, are already proposed on cfDNA by Guardant 360 (Guardant Health, Redwood City, CA, U.S.A.) or FoundationOne Liquid CDx (Foundation Medicine, Cambridge, MA, U.S.A.). Guardant 360 detects point mutations (74 genes), copy number amplifications (18 genes), fusions (six genes) and small insertions or deletions (three genes) [163]. The FoundationOne Liquid CDx panel (300 gnes) detects base substitutions, insertions/deletions, copy number alterations and genomic rearrangements as well as determining the blood tumor mutational burden (TMB) and microsatellite instability status [164].

The technical cost and turn-around time from biopsy to molecular results are a crucial point in the clinical practice: NGSs can require from 7 to 15 days, sometimes more if the sequential approach or test execution in external sequencing platforms are needed. Moreover, some NGS applications remain expensive, and the sample quality threshold is generally high. In this context, it’s important to challenge NGS with other multiplex techniques available for genetic alteration analysis that could improve the workflow analysis.

#### 3.2.2. Non-NGS Multiplex Targeted Methods

These non-NGS multiplex-targeted methods allow for the genotyping of well-known, validated hotspot mutations or genetic alterations. Their use for the characterization of resistance mechanisms has to be evaluated, as the interpretation of the molecular results from these tests is only limited to the list of alterations tested.

NanoString technology can be performed to detect gene fusions and is adequate for the analysis of degraded clinical samples [165,166,167].

Another non-NGS multiplex method uses the iPLEX chemistry and matrix-assisted laser desorption ionization time-of-flight analysis on a MassARRAY mass spectrometry platform [168]. It uses panels to investigate the presence of relevant mutations (in *BRAF, EGFR, ERBB2, KRAS* and *PIK3CA) on DNA extracted from FFPE tissue samples or cfDNA* as well as the presence of rearrangements (*ALK*, *ROS1* and *RET)* on RNA [169]. As described elsewhere, the MassARRAY system enable fast, low-input-adapted and cost-effective genotyping for a targeted set of mutations [170].

#### 3.2.3. Sensitive PCR-Based Methods

Non-multiplex techniques are less and less used for molecular testing after relapse following EGFR TKI in NSCLC, as molecular mechanisms are complex and numerous. The only exception concerns *EGFR* T790M mutation detection in tumor or cfDNA at progression after treatment with an EGFR TKI of the first–second generation. Nevertheless, such very sensitive techniques could be useful to detect mutated subclones.

Digital PCR techniques, such as BEAMing (beads, emulsion, amplification, magnetics) and droplet digital PCR (ddPCR) are substantially highly sensitive (0.1% to 0.01%) with a very short turnaround time (two days); thus, they are particularly adapted for cfDNA analysis [171]. Finally, the recent development of multiplex ddPCR readouts, which combine both fluorescence color and intensity, has allowed for a relatively low level of multiplexing. Nevertheless, this technique has to be evaluated in clinical situations of such acquired resistance to EGFR-TKIs [172].

Molecular evaluation at initial diagnosis and resistance are two very different clinical situations, which must be differently analyzed by appropriate and dedicated molecular techniques [173,174,175,176]. At resistance, and from a clinical perspective, targeted DNA NGS panels combined with RNA NGS panels seem to be the best molecular approaches that are used today more on tumor than on blood samples.

## 4. Molecular Testing and Resistance to EGFR-TKIs

All patients with NSCLC tumors harboring *EGFR*-activating mutations treated by EGFR-TKIs ultimately develop resistance. Resistance to EGFR-TKIs depends on modifications of the initial tumor tissue with different possibilities: increasing of the presence of sub-clonal populations with intrinsic resistance to TKIs under therapeutic pressure, tumor mutability with *de novo* molecular alterations, or tumor capacities to develop adaptive resistance by histological transformation. The intra-patient heterogeneity as well as multiple co-alterations in the same tumor are also challenging for counteracting tumor progression by biological and treatment strategies.

The relatively high frequency of oncogene fusions at resistance, usually extremely rare at diagnosis, requires exploration. This high frequency of acquired fusions supports a predisposition for genomic rearrangements driven by the selective pressure of osimertinib. Some of these fusions seem targetable with some therapeutic options for the patients with a tumor harboring such alterations.

The variable sensitivity of the molecular tests must be taken into account, on one hand, to ensure the detection the mechanism of resistance, and on the other hand, to avoid false positive results or false alterations without clinical implications.

### 4.1. Anticipation of Resistance Mechanism at Diagnosis

#### 4.1.1. Sub-Clonal Alterations at Diagnosis

Development of resistance after a short period may also result from preexisting sub-clones that emerge quickly on treatment or later among a drug-tolerant tumor. The selection of sub-clonal alterations at resistance under EGFR-TKIs could be related to EGFR on-target (i.e., T790M mutation) or off-target mechanisms. The question is to know whether the detection of such sub-clonal alterations in a tumor biopsy at baseline before progression will effectively correspond to the main resistance mechanism at the time of progression.

##### EGFR T790M Mutations

If the selection of T790M mutation after first–second-generation TKI is no longer relevant after third-generation EGFR-TKI, which is now largely administrated in first-line treatment, the comprehension of the biological mechanism of such mutations could help to understand other mechanisms of resistance. The natural history of *EGFR* T790M mutation appears to be highly complex. T790M mutations could be involved earlier as the selection of pre-existing T790M mutation or later among a drug-tolerant tumor.

While T790M represents a good post first–second-generation EGFR TKI prognostic factor, there are contradictory results concerning the predictive/prognostic value of baseline pre-treatment somatic T790M sub-clones [32,176]. Pre-treatment tumor T790M mutations were reported to occur with varying prevalence, ranging from <1%, using the Sanger technique, to 80%, using more sensitive allele-specific techniques. The detection of the T790M mutation in FFPE samples at a low allelic frequency is challenging due to the fact that it is a C > T transition and mimics the FFPE artefact. Data on pre-treatment T790M mutation frequency proved discordant, even when using the same molecular techniques, probably due to the choice of different thresholds. An ancillary analysis of the French Biomarker Study with quantification of pre-treatment tumor T790M mutation using ultra-sensitive droplet digital PCR identified this mutation in 8% (19/240) of cases [32]. T790M-positive and T790M-negative populations were not different for clinical baseline characteristics. A negative prognostic value under first-generation EGFR-TKIs was established only for T790M VAF over 1%.

##### Other Alterations

*MET* amplification is very rare (<5%) in EGFR-TKI naïve patients, with a variable frequency increasing from 5–10%, 5–50% to 7–15% at resistance after first/second-line, second-line and first-line treatments of third-generation EGFR-TKIs, respectively [58,63,69,70] (Figure 2).

No data are available concerning sub-clones of other gene mutations/amplification/fusions before EGFR-TKIs treatment.

#### 4.1.2. Histological Transformation

There are reliable biomarkers that predict high risk of histologic transformation. Different biomarkers seem to predict histological transformation, from NSCLC to SCLC tumors, such as loss of *EGFR* mutations, inactivated mutation of *TP53*, inactivated mutation of *RB1*, and activating mutation of *PI3KCA* genes [117,118,177,178]. Nevertheless, these mutations are not enough to induce histological transformation; additional factors seem necessary, such as *MYC* and *BCL2* overexpression and *AKT* over-activation [31].

#### 4.1.3. Molecular Characterization of Residual Tumor Cells after Partial Response or Stable Disease

There is a spatial and temporal heterogeneity of resistance acquired under different lines of EGFR-TKI that seems to increase with new TKIs. In-depth characterization of the “persistent” cell population of therapy-induced drug tolerance [179,180] through an on-treatment biopsy may help to identify the precursors destined to drive eventual resistance, and potentially allow for the introduction of early, tailored, combination therapies capable of eradicating the sub-clones before the point of clinical progression [111]. Drug-tolerant cells appear to be slow-cycling, epigenetically reprogrammed, but poorly characterized. They have senescent-like features and can escape from senescence at time of progression. The identification of new pathways associated with drug tolerance (RhoB) was recently identified [81]. Drug tolerance could be reversed by HDAC inhibitors, by IGF1R inhibitors, AKT inhibitors, GPX4 inhibition, NOTCH inhibition or YAP inhibition [181,182]. In this perspective, another treatment strategy could be to combine the targeted therapy with chemotherapy.

### 4.2. Molecular and Histological Characterization Identification at Time of Resistance

#### 4.2.1. Plasma Cell-Free DNA

##### Physiopathology

Cell-free DNA (cfDNA) is shed into the bloodstream by many (but not all) solid tumors and comprises a small portion of the total plasma cfDNA [151]. cfDNA is produced by cell apoptosis, necrosis, or active excretion and circulates in the blood (plasma). cfDNA has the potential to identify oncogenic drivers in tumor-derived DNA present in blood and capture intra-tumoral heterogeneity not addressed by biopsy of a single site, potentially obviating the need for invasive tissue biopsies in some cases [183].

Additionally, cfDNa analysis could be helpful when molecular analysis of tumor tissue suffers failure [184]. cfDNA is easily obtained through minimally invasive blood sampling and can be a specific and sensitive biomarker for the detection of *EGFR* mutations or other molecular alterations in patients whose tumors shed DNA [153,185,186].

In addition to the limitations of the plasma genotyping assay for fusion detection, intrinsic disease characteristics can compromise the utility of plasma genotyping. For example, studies have shown that the cfDNA yield is lowest when metastatic sites are limited to the thoracic cavity or CNS [29]. Larger tissue plasma concordance studies are needed to determine whether the relatively low sensitivity is truly reflective of all patients or rather represents a population inadvertently enriched for those with lower disease burden.

At initial diagnosis of non-squamous NSCLC, *EGFR* mutation testing is recommended, using tumor tissue biopsies. In some clinical settings in which tissue is limited and/or insufficient for molecular testing, physicians may use a plasma circulating tumor DNA assay to identify EGFR mutations. The situation is different at progression.

##### Technical Considerations

The genotyping of plasma DNA that have enabled “liquid biopsies”, is distinct from the molecular analysis of circulating tumor cells, which can be more technically challenging. Genomic analysis of plasma DNA has been possible for over a decade but has only become more clinically relevant with the emergence of molecularly high sensitivity approaches, which now permit the high-sensitivity detection of cfDNA in plasma as low as an allelic fraction (AF) of 0.1%. Modern cfDNA genotyping approaches include both single-gene PCR-based assays as well as multigene NGS-based assays.

The comparison between a tumor and cfDNA analysis needs to have the smallest time interval and no treatment between the two samples because the tumor shedding of cfDNA may vary over time [187]. Further, targeted therapies of the immune response may allow clearance of cfDNA fragments from plasma with molecular monitoring. Tumor heterogeneity is described as a potentially important source of discordance in the number of detected alterations between tumor tissue genotyping and the plasma cfDNA genotyping. Such heterogeneity appears to be less relevant to the initial genotyping of NSCLC in which driver alterations are largely truncal and present across all sites of disease. A substantial majority of targetable driver alterations, including EGFR, are clonal. Genomic heterogeneity is particularly apparent after drug resistance. Furthermore, the sensitivity of cfDNA-based assays is related to the extent of metastatic spread and is increased in patients with extra-thoracic metastases, particularly in those with liver and/or bone metastases [71].

*Non-NGS Techniques*

Using the reference central Cobas tissue test results of the FLAURA trial, positive percent agreements with the Cobas plasma test results for *EGFR* Del19 and L858R detection were 79 and 68%, respectively, supporting the utility of Cobas tissue and plasma testing to aid the selection of patients with *EGFR* mutated advanced NSCLC for first-line osimertinib treatment [188]. cfDNA genotyping in lung cancers, using digital PCR (dPCR) for detecting *EGFR* mutations, showed 100% specificity for the plasma detection of drivers with a sensitivity depending on the cut-off for positive cases [129,189,190].

*Targeted NGS*

Studies have shown that NGS can be used to detect actionable gene mutations with high accuracy in plasma samples, and new targeted NGS methodologies are being developed that improve the sensitivity and the specificity in cases such as samples with low VAF [191]. Orthogonal comparison of four plasma NGS tests (panels of 20 to 60–70 genes) suggests that technical factors are a major source of assay discordance, and to a lesser extent, biological factors, such as clonal hematopoiesis and tumor heterogeneity [121]. A recent study showed that ultra-deep plasma NGS with clonal hematopoiesis filtering results in new targeted oncogenic drivers and resistance mechanisms in patients with NSCLC, including when tissue biopsy is inadequate for genotyping, with a sensitivity of 75% [192]. False-negative factors are largely associated with low tumor DNA shed below an assay technical limit of detection. False-positive result are attributed to genomic heterogeneity of the tumor, particularly in the setting of acquired drug resistance [122].

The concordance analysis of AURA3 trial cfDNA samples analyzed by Cobas, ddPCR and targeted NGS (Guardant360) demonstrated a strong correlation between the NGS and ddPCR tests, and all discordant samples had allelic fraction ≤1%; this suggested that the enhanced sensitivity over the Cobas plasma test could be a true test effect [92]. For the discordant results between ddPCR and NGS samples, the majority were near the limit of detection of both assays and were supported by low read numbers. Re-testing samples that were negative for ddPCR via increased DNA input resulted in changes to a positive status in more than half of the samples. These data suggest that the detection of low levels of T790M relies on a greater input DNA amount to maximize signal-to-noise ratios.

A high specificity and a high positive predictive value was first established for the Cobas and ddPCR approaches, even at extremely low VAFs. This high level of specificity was replicated with targeted NGS -based assays. The sensitivity of Cobas or the NGS-based assay is high (68–79%), but this imperfect level of sensitivity is believed to be related to variations in the amounts of cfDNA shed into plasma, with an understandable decline in sensitivity in the presence of negligible amounts of cfDNA. Nevertheless, PCR-based tests are more accessible, have a shorter turnaround time and lower cost, and require a smaller sample size, compared with NGS. Other factors that can influence the selection of a test include reimbursement and mutation prevalence in the target population. In cases where there is insufficient tissue or DNA in the plasma, single-gene testing could be a useful alternative to screening for multiple mutations.

Plasma cfDNA-targeted NGS detected a variety of oncogenic drivers with a shorter test around time (TAT) compared with tissue NGS. Positive findings on plasma NGS were highly concordant with tissue NGS. However, the negative finding in plasma requires further testing [124]. In another hand, in patients unable to have tissue NGS, plasma cfDNA-targeted NGS increased the number of targetable mutations. The plasma-based targeted mutation VAF had no correlation with the depth of the RECIST response [193].

Using cfDNA analysis is different at diagnosis, compared to progression time, and its utility varies among the generation of EGFR-TKIs and lines of therapeutics. cfDNA is easy to repeat and is a possibility in the early detection of subclonal resistance mechanisms, which may be useful to guide patient management and future drug development.

##### Place of cfDNA Analysis at Progression

*EGFR Mutations under First–Second-Generation EGFR-TKIs*

The principal mechanism of resistance at progression after first/second-generation EGFR TKI is second *EGFR* mutation T790M, present in half of the cases. This alteration can be easily tested in cfDNA by targeted techniques, PCR-based or ddPCR, or by NGS technologies.

It can replace tumor genotyping only if the plasma test is positive. A reflex tumor tissue test should be performed after a plasma T790M negative test result. Repeated tumor biopsies as well as plasma genotyping at the time of progression are crucial steps in unravelling resistance mechanisms and guiding future treatments.

Previous studies have shown that the T790M resistance mutation is more easily detected in the cfDNA of patients with metastatic disease versus a locally advanced disease, because the detection of T790M mutation in plasma relies on the presence of adequate copies of the *EGFR* gene being shed in cfDNA, which may correlate with the disease burden [115]. More patients with extrathoracic disease (M1b) had detectable T790M in their plasma in comparison with those without extrathoracic disease (M stages 0–1a). It is a correlation between the baseline tumor size and detectable cfDNA in both the concordance analysis and the NGS shedder-versus-non shedder analysis.

*EGFR Mutations under Third Generation EGFR-TKIs*

Data are available concerning cfDNA analysis of patients with T790M positive tumor tissue at baseline, treated by second-line osimertinib. Some evidence suggests that the T790M clonality level at baseline, i.e., the size of the T790M-positive population of tumor cells, might influence the response to third-generation EGFR-TKIs. It is also the case for T790M sub-clonality.

In a retrospective analysis of cfDNA from patients from AURA3 study, patients with a cfDNA baseline T790M-negative status had prolonged PFS and fewer progression events in comparison with patients with a plasma T790M-positive status, regardless of treatment arms [92]. This result suggests that a detectable cfDNA T790M mutation could be a reflection of the disease burden, an association between the baseline tumor target lesion size and the shedding status, and could be a prognostic factor. Patients who are not cfDNA positive for either the activating and resistance *EGFR* mutations may have smaller tumors. Regardless of the NGS or Cobas T790M plasma status, clinical outcomes were consistently improved in osimertinib-treated patients in comparison with those receiving platinum–pemetrexed [92]. This is as expected because all patients were tissue T790M positive, which is consistent with the overall AURA3 result [60]. This finding is supported by results from a recent smaller study in which patients receiving osimertinib with low levels of T790M in cfDNA had improved PFS and OS in comparison with patients with high levels; low levels of T790M and complete clearance after 2 months were also associated with better outcome [194].

A recent study retrospectively analyzed baseline plasma from patient enrolled in the AURA3 clinical study to identify patients with subclonal T790M (i.e., the presence of T790M in only a small fraction of tumor cells) and response to second-line osimertinib [31]. At baseline, approximately 66% of patients had both activating *EGFR* and T790M mutation in cfDNA, 10% were positive for either the activating *EGFR* or T790M mutation, and 25% had no detectable *EGFR* mutation and were considered non-shedders. In this study, NGS analysis of baseline cfDNA showed a median VAF at 5.9% for ex19 and L858R mutations and at 2.3% for T790M mutation, which was significantly lower (*p* < 0.001). The relative T790M VAF values (T790M VAF value related to the activating EGFR mutation VAF) were highly variable with a median of 37.7%. T790M subclonality at baseline was defined with a relative T790M value under 30% (below the median) and T790M clonality over 30% [24,31]. The median tissue T790M clonality value was comparable to the median plasma value, the discrepancy clonality result between plasma and tissue NGS being possibly associated with tumor heterogeneity, which is better captured by plasma NGS [31]. Osimertinib treatment appeared superior to chemotherapy, independent of the T790M clonal status. Interestingly, subclonal T790M genotype was associated with shorter PFS, and less responders under second-line osimertinib and under chemotherapy, suggesting that other non-T790M alterations could co-occur in these tumors [31].

T790M subclonal mutations were enriched for co-occuring activating PI3KCA mutations, which was demonstrated to reduce sensitivity to osimertinib in EGFR-mutant cell lines.

Longitudinal cfDNA profiling with status of T790M at progression could also be interesting. Outgrowth of a TKI-resistant clone after clearance of the T790M-positive subclone was associated with shorter mPFS in NSCLC patients who tended to have a smaller fraction of T790M over the activating EGFR mutation in their tumor at baseline [30,109,110]. Detection of T790M subclonality in pre-treatment plasma could lead to liquid biopsy monitoring to detect resistant clones early.

Identification of primary resistance in second-line treatment could also depend on the area of biological testing realized at the time of resistance after first–second-generation EGFR-TKIs. For example, SCLC transformation was described as a putative mechanism of primary resistance to osimertinib in five cases, only tested in cfDNA by ddPCR for T790M analysis [86]. In such cases, a low ratio (lower than 0.03) between T790M and *EGFR* activating mutation in the blood was detected before osimertinib treatment. For three patients, the *EGFR* mutational analysis was T790M-negative when re-assessed by using a less sensitive method (therascreen) on the same liquid biopsy sample analyzed by ddPCR before osimertinib therapy. Although liquid biopsy is a relevant tool to diagnose T790M presence in NSCLC patients resistant to EGFR-TKI, in the case of low ratio T790M/activating mutation, tissue biopsy should be considered to exclude the presence of SCLC transformation and/or other concomitant resistance mechanism.

Other data are available concerning cfDNA analysis of patients with *EGFR* positive tumor tissue at baseline, treated by first-line osimertinib. At baseline, patients included in the FLAURA study were required to have tumor tissue *EGFR* mutated advanced NSCLC, and to have blood samples for retrospective central Cobas plasma cfDNA analysis of the *EGFR* status. In both treatment arms, PFS was prolonged in baseline plasma cfDNA EGFRm-negative (23.5 and 15 months) versus positive patients (15.2 and 9.7 months), potentially due to the patients having lower tumor burden [188]. Future cfDNA plasma testing with identification of *PI3KCA* pathway alterations could also be interesting, as PI3KCA mutations were described to be acquired in cfDNA of the FLAURA study [58,62,195] and could classify the patients for a combination treatment of osimertinib and PI3K pathway inhibitors.

cfDNA analysis gained popularity in an era when EGFR T790M was the main driver of resistance to older TKIs. T790M can be easily detected in the plasma and bears valuable information about clonal and subclonal population evolution during second-line osimertinib. cfDNA, at the time of resistance after second- or first-line oismertinib treatment, must be able to detect various *EGFR* and non-*EGFR* mutations. Furthermore, cfDNA alone cannot detect histologic changes. cfDNA is, therefore, insufficient in patients progressing on first-line osimertinib, where T790M is not present and various molecular alterations and histological transformations are not uncommon (Figure 4).

Analysis of cfDNA had also differential sensitivity for copy number changes and chromosomal rearrangements, compared with tissue analysis [121,122,123,125].

*Other Molecular Alterations, Copy Number Variations and Gene Fusions*

Copy number variations (CNV) are challenging to detect and accurately quantify using cfDNA NGS, and events could be missed. Amplification is better in tissue analysis by NGS rather than plasma. In a study of temporally matched cfDNA and tissue samples of NSCLC patients with evidence of cfDNA in their blood, only 6% of amplification were detected in cfDNA. Detection of gene amplification can be difficult, particularly when the cfDNA content is low [136]. Less robust detection of amplifications in samples with lower cfDNA fraction was observed in multiple solid tumor studies with the use of various assays, suggesting that it may be a limitation generally for cfDNA testing [136].

Analysis of cfDNA is a promising strategy for identifying gene fusion at resistance to EGFR-TKIs. However, detection of rearrangements in plasma is more challenging than identifying short variants, such as point mutations and indels. Sensitivity for gene fusion detection might also be affected by technical factors [125,196]. Noting the heterogeneity of fusion sequences observed in NSCLC, it could be hypothesized that the difference in hybrid-capture techniques and bioinformatic calling (Guardant360^®^, ctDX-Lung) may be a source of variations in sensitivity among these assays [125]. Most discordant cases between plasma assays are at low Afs, as they are especially susceptible to stochastic differences. Early studies suggest that plasma genotyping assays detect *ALK* fusions with a high degree of concordance with tissue genotyping [24,163,197]. For *ROS1* fusion, the variety of breakpoints and number of fusions partners is challenging for DNA capture-based plasma genotyping (Guardant360 NGS assay) [196]. Sensitivity of plasma genotyping for detecting *ROS1* fusion was 50% and ALK fusion was 86% in cohorts of patients with known gene fusions, relapsing on targeted therapy [196,197]. A recent study based on targeted amplicon-based assay cfDNA analysis with a 36 genes panel was able to detect *ALK/ROS1* fusion in 67% of cases, with a higher detection for *ALK* fusions at TKI failure [198].

It is important to note that there was no study about cfDNA fusion detection at progression under TKI-EGFR, situations known to be associated with different breakpoints or singular partners of fusions. Plasma assays are DNA-based and the sensitivity of plasma genotyping for fusion-driver subsets is highest when there is a conserved breakpoint (i.e., *ALK*). NGS-based plasma genotyping could be an informative method for identifying fusions but in cases where no fusion is detected in the plasma, tissue analysis should be performed. Fusion detection is better in RNA tissue analysis, which is better than DNA tissue analysis, which is better than plasma NGS analysis. As a result, plasma genotyping may not be a universal solution for overcoming the tissue genotyping delay [199].

*Non-Shedder Patients; Different Clinical Situations*

EGFR non-shedders have a smaller baseline tumor target lesion size, the fewest detectable genomic alterations and the lowest VAFs of these mutations [31,92]. Previous studies have shown that levels of cfDNA shedding into plasma correlate with the tumor burden, and the lack of detectable cfDNA early in EGFR-TKI therapy is associated with better clinical prognosis.

In the FLAURA study, patients with “nonshedding” tumors seemed to have a better prognosis than patients with detectable cfDNA, as in the AURA study and pooled analysis of the AURA extension/AURA2. The improved PFS in patients with negative plasma results may be due to lower tumor burden.

*Histological Transformation*

Although liquid biopsy is a relevant tool to diagnose T790M mutation presence in NSCLC patients resistant to first/second generation EGFR-TKIs, in the case of low ratio T790M/activating *EGFR* mutation, tissue biopsy should be considered to exclude the presence of SCLC transformation and/or a concomitant resistance mechanism [86]. NSCLC patients who harbor inactivated *RB1* and *TP53* may warrant monitoring for histologic transformation into SCLC during their clinical history [117,118]. The assessment of *RB1* and *TP53* mutational status on cfDNA as well as the test of neuron-specific enolase (NSE) levels in plasma at the time of progression could be taken into account to unravel a potential SCLC transformation [86]. The critical role of tissue sampling appeared in the evaluation of patients who progressed on second-line as well as on first-line osimertinib treatment [70].

#### 4.2.2. Tissue Analysis

In situations of progression under EGFR-TKI, the better tumor sample to be used for molecular/histological analysis seems to be from the site of progression---tissue or cytological samples (i.e., pericarditis, pleural, and bronchoalveolar lavage)---if containing enough tumor cells. Bone biopsy could be used if tumor cells are present in the sample. As for initial molecular testing, in the case of tissue samples, the pathologist realizes a histological diagnosis and selects the best sample for molecular analysis.

NGS testing provides the prevalence of established baseline mutational events co-occurring with T790M patients of the AURA3 trial with *TP53* mutations (64%) and *EGFR* amplification (33%) and other studies [200]. This study did not show any significant association between co-occurring mutations (*PI3KCA*, *TP53* and *MET/HER2* amplification) and a poor response rate or reduced clinical benefit, but had a small sample size of each genomic alterations [69].

In the FLAURA trial, the local Cobas tissue test results were retrospectively confirmed EGFR mutation positive (for Del19 and L858R mutations) by central analysis in 97% of the patients [188]. A similar sensitivity result (99%) between the Cobas tissue test and local testing methods for the detection of ex19del (99%) or L858R mutation (95%) was observed in a study that analyzed samples obtained from patients randomized to the expansion cohort of the AURA phase I trial and the pooled phase II AURA extension/AURA2 studies [29]. The proportion of patients with uncommon mutations detected in their tissue samples (2%) was slightly lower than other reports from larger studies, in which the range was typically 10–18%.

In situations of relapse, NGS analysis must be realized by wider DNA NGS panels in order to detect all mutations (substitutions, little deletions, insertions or indel) and copy number variations of different genes related to resistance (Figure 4).

At the same time, it is recommended to test the sample by RNA NGS fusion panels, as some mechanisms of resistance involved targetable gene fusions (Figure 2, Figure 3, Figure 4). As biopsy at progression often consists of a small tumor sample, NGS RNA panels analysis appears better than multiple IHC/FISH analysis. As a result of incomplete coverage of the introns that house splice sites and fusion breakpoints, RNA-based approaches seem better for detecting the fusion transcript and MET exon 14 skipping, compared to DNA NGS analysis [199,201]. A parallel approach of combining DNA/RNA NGS seems to be the most efficient strategy (Cohen D, JTO 2020) and provides particular flexibility in the constantly evolving landscape of resistance mechanisms of successive targeted drugs. Extracting the total nucleic acid at a single time point can improve the yield of NGS relative to independent extractions for DNA and RNA testing.

The delay of results of such an analysis must be compatible with clinical care [1,202].

## 5. Perspectives

Two strategies to overcome acquired resistance under first generation EGFR TKIs without T790M mutation or under third generation EGFR TKIs (second or first line) can be proposed: the first strategy is dependent on the molecular biology results in the case of identification of a specific targetable mechanism of resistance; the second strategy is not dependent on the molecular results in the case of multiple mechanisms or no mechanism of resistance. The challenge is to know whether the same efficacy as that for naïve NSCLC patients with the same biomarker could be observed in the context of acquired resistance. Moreover, knowing the molecular mechanisms underlying EGFR-TKIs’ acquired resistance should also be helpful in order to prevent the emergence of those resistances.

### 5.1. Biomarker Driven Approaches

#### 5.1.1. EGFR-Dependent Mechanisms

Fourth-generation EGFR-TKIs (against EGFR C797S and T790M mutations) are in development with in vitro and in vivo activity, alone or combined with osimertinib (EAI045; JBJ-04-125-02); BLU-945) [89,203,204,205], but there is no available clinical trial. The combination of osimertinib with first-second generation EGFR TKIs in cases with C797X and T790M in trans could be effective as well as the use of first–second-generation EGFR TKIs in cases with C997X and the absence of the T790M mutation [68,104,117]. Preclinical models suggest the effectiveness of EGFR-TKI with cetuximab or brigatinib with anti-EGFR antibody, but clinical applications have yet to confirm them [89,206,207,208].

#### 5.1.2. EGFR-Independent Mechanisms

##### MET Amplification

MET amplification is one of the most frequent EGFR-TKI mechanisms, more often after third-generation EGFR-TKIs.

Savolitinib, a MET TKI combined with osimertinib, showed ORR at 33% with a median PFS of 5.4 months, which is lower than that in EGFR-TKI naïve patients [89,209]. Phase II is ongoing. Capmatinib, another MET TKI combined with gefitinib, showed ORR at 27%, and reached 47% in cases with a MET gene copy number ≥6, with tolerable toxicity [89,210]. Tepotinib, another MET TKI combined with gefinitib, showed a higher ORR, compared to chemotherapy [89,211]. Tivantinib is another oral selective non-ATP-competitive MET inhibitor that has been described as a promising targeted therapy combined with erlotinib [212]. On the other hand, combining crizotinib and erlotinib never reached phase II studies because of the amplified toxicity [213].

Bispecific EGFR and MET antibody (amivantanab) are also emerging in those treatment strategies combined with another EGFR third-generation TKI (lazertinib) and will be developed in the phase II clinical trial [214].

##### HER2 Amplification

The presence of HER2 overexpression suggests a lower sensitivity to third-generation in T790M-positive EGFR-mutant NSCLC; it would seem logical to combine osimertinib and anti-HER2 antibodies. In pre-clinical studies, trastuzumab–emtansine (T-DM1) showed some promising results in cases with HER2 amplification and EGFR mutation, which previously failed in EGFR-TKI treatment [89,215,216]. T-DM1 monotherapy showed some activity in such situations [89,215]. T-DM1, combined with another pan-HER TKI (neratinib), showed tumor regression in pre-clinical models and warrants further studies [215,217].

##### Other TKIs

The combination of osimertinib with other specific TKIs, according to the identification of the molecular mechanism of resistance under EGFR-TKIs (AXL, AKT, MEK, BRAF, CDK4-6), was reported in pre-clinical studies, but very few clinical cases are reported [89,128,218]. The ORCHARD phase II trial, using a biomarker strategy with a combination of osimertinib with different TKI (savolitinib, gefitinib, necitumumab), is ongoing [89,219].

##### Fusions

If there are rare reports of acquired fusions at progression under EGFR-TKIs, the incidence of such alterations seems to be more important than in naïve NSCLC patients, but with unusual partners of fusions and/or breakpoints. Incidence seems to be increasing with the generation of EGFR-TKIs. It is unknown whether this potential enrichment of acquired fusions is related to the more potent EGFR inhibition of osimertinib or to the later line settings after the other line of EGFR-TKIs. The efficacy of the combined treatment of osimertinib with alectinib, crizotinib, trametinib or encorafenib, was reported in clinical cases [116,132,133].

### 5.2. No Biomarker-Driven Approach

In all the other situations—with other biomarkers, combined biomarkers or no biomarkers—a no-biomarker-driven approach must be tested.

#### 5.2.1. Chemotherapy, with or without EGFR-TKI Continuation

In the absence of an identified molecular target, chemotherapy still seems to be the best strategy. In the IPASS study [37], patients who had received gefitinib in the first treatment line and chemotherapy in the second line had similar overall survival results; patients benefitted from chemotherapy before gefitinib, suggesting, therefore, the efficacy of chemotherapy after EGFR-TKI failure [72]. Continuing TKI treatment with the addition of chemotherapy can be an option, even if it is controversial. Indeed, phase II or III trials of the combination of chemotherapy with gefitinib or erlotinib failed to show benefits in PFS and were even associated with worse OS, even if the combination appeared to be generally tolerable [214,220].

#### 5.2.2. Immunotherapy

Immunotherapy with monoclonal antibodies against programmed death-1 (PD-1) and programmed death ligand-1 (PD-L1) but also anti-CTLA4, recently drastically improved survival and quality of life of NSCLC patients. *EGFR* mutation status was shown as associated with PD-L1 expression [221]. Nevertheless, the role of immunotherapy is minimal in EGFR-mutated NSCLC cases. Patients harboring EGFR-sensitizing mutations appeared to be less sensitive to immune checkpoint inhibitors, such as monotherapy [222]. Those results also appeared in the IMMUNOTARGET registry, where EGFR-mutation-positive patients treated with immunotherapy showed worse PFS results [223]. The poor clinical response rate of patients with EGFR-mutant NSCLC to anti-PD-1/PD-L1 treatment mechanisms is still unclear but may be associated with the low tumor mutational burden (TMB) of those cancers, leading to weak immunogenicity [224]. Uncommon *EGFR* mutations (such as G719X, L861Q, S768I, and Exon 20 insertion) would then show a more favorable response to PD-1 inhibitors [225]. The IMpower150 trial reported better OS and PFS with atezolizumab, bevacizumab, carboplatin and paclitaxel, compared to bevacizumab, carboplatin and paclitaxel in patients with non-squamous NSCLC, including those with *EGFR* mutations, and those who had received prior TKIs [226]. Recent studies are, therefore, trying to combine chemotherapies and immunotherapy in order to increase sensitivity to immunotherapy by modulating the tumor microenvironment for patients with EGFR-mutant NSCLC [89]. Toxicities seem, nevertheless, to be a limiting factor for those associations.

#### 5.2.3. Other Treatments

Because HER3 is frequently overexpressed in EGFR-mutant tumors, HER3-directed antibody-drug conjugates were studied. Patritumab deruxtecan, therefore, showed promising response rate results for patients after EGFR-TKI failure, regardless of the presence or absence of other specific mutations previously listed [227]. As TROP2 (trophoblast cell-surface antigen 2) is also often overexpressed in NSCLC and associated with poor outcomes [228], anti-TROP2 treatments are currently being tested as an actionable biomarker for those patients in TROPION-PanTumor01.

## 6. Conclusions

Despite significant advancements in the treatment of EGFR-mutant NSCL, the development of resistance remains a universal challenge. The biology of resistant tumor cells grows increasingly complex as our targeted agents become more potent. It is becoming clear that simply sequencing a series of EGFR-TKIs is not an effective long-term strategy.

Understanding the complexity of EGFR-TKI resistance mechanisms must be a priority in clinical practice to better develop new strategies [229]. The best samples to be analyzed at progression under third-generation EGFR-TKIs or after negative analysis of cfDNA under first–second-generation EGFR-TKI remain tumor tissues; even though some progress was made for cfDNA analysis, it is generally not accessible in clinical practice [230]. Another approach could be the identification of patients who will not have a durable response due to the presence of *TP53* mutation, or combination of *TP53* and *RB1* mutations or lack of plasma clearance of *EGFR* mutation by dynamic plasma cfDNA monitoring. Finally, eradicating the seeds of resistance before progression occurs is necessary to make a meaningful impact on long-term patient outcome. New-generation sequencing is essential to overcome EGFR-TKI resistance [231].

## Figures and Tables

**Table 1:** Studies of targeted therapies in first-line therapy for advanced NSCLC with *EGFR* mutation.

Generation EGFR-TKI	Study	Agent	*EGFR* Mutation	*N*	Median PFS (Months)	PFS HR [CI]
First generation	IPASS [33]	Gefitinib	Del19/ L858R	261	9.5 vs 6.3	0.48 (0.34–0.67)
	NEJGSG002 [34]			228	10.8 vs 5.4	0.30 (0.24–0.44)
	WJTOG3405 [35]			177	9.2 vs 6.3	0.49 (0.38–0.72)
	OPTIMAL [36]	Erlotinib	Del19/ L858R	154	13.1 vs 4.6	0.16 (0.10–0.26)
	EURTAC [37]			173	9.7 vs 5.2	0.37 (0.25–0.54)
	ENSURE [38]			217	11.0 vs 5.5	0.34 (0.22–0.51)
	CONVINCE [39]	Icotinib	Del19/ L858R	285	11.2 vs 7.9	0.61 (0.43–0.87)
Second generation	LUX-Lung 3 [40]	Afatinib	Del19/ L858R	345	11.1 vs 6.9	0.58 (0.43–0.78)
	LUX-Lung 6 [41]			364	11.0 vs 5.6	0.28 (0.20–0.39)
	ARCHER 1050 [42]	Dacomitinib	Del19/ L858R +/- T790M	452	14.7 vs 9.2	0.59 (0.47–0.74)
Third generation	FLAURA [10]	Osimertinib	Del19/ L858R	556	18.9 vs 10.2	0.46 (0.37–0.57)
Others EGFR ins20 inhibitors	ZENITH20-cohort1 [43]	Poziotinib	EGFR/HER2 ins20	88	4.1	
	Phase I/II [44]	Mobocertinib	EGFR ins20 EGFR ins20	70	7.3	
	CHRYSALIS [45]	Amivantamab		50	8.3	

**Table 2:** Molecular technics.

Technique: Multiplex Strategy	LoD	Sample	M	CNV	Trsl	Exp	Use
High throughput							
WGS-WES	5%	Frozen	XX				R
WTS-RNAseq	5%	Frozen	XX		X		R
Targeted NGS							
DNA							
FoundationOne CDx^®^	2–5%	FFPE	XX	(X)	X		R, C
FoundationOne Liquid CDx^®^	0.2–1%	cfDNA	XX	(X)	X		R, C
Guardant Assay^®^	0.2–0.4%	FFPE, cfDNA	XX	(X)	X		R, C
Enlarged custom targeted panels	2–5%	FFPE, cfDNA	XX	(X)			C
RNA							
TruSight RNA fusion panel^®^ (Illumina)	10–15%	FFPE	X		XX	X	C
Targeted RNAscan custom^®^ (Qiagen)	10–15%	FFPE	X		XX	X	C
Oncomine Focus^®^ (Thermo Fischer)	10–15%	FFPE	X		XX	X	C
FusionPlex kit^®^ (ArcherDx)	10–15%	FFPE	X		XX	X	C
Technique, targeted methods	LoD	Sample	M	CNV	Trsl	Exp	Use
Nanostring^®^	10000 transcript copies	FFPE	X		X	X	R
Real-time PCR (Cobas^®^, Therascreen^®^)	1–5%	FFPE, cfDNA	X			X	C
Idylla^®^	5%	FFPE, cfDNA	X				C
Mass-Array^®^	1–2%	FFPE, cfDNA	X				C
dPCR	0.1–0.01%	FFPE, cfDNA	X	X			R, C

NGS: new generation sequencing; WGS: whole genome sequencing; WES: whole exome sequencing; WTS: whole transcriptome sequencing; dPCR: digital PCR; FFPE: formalin-fixed paraffin-embedded; cfDNA: cell-free DNA; LoD: Limit of Detection; M: mutation; CNV: copy number variation; Trsl: gene translocation; R: research; C: clinics.
